# Surface or Internal Hydration – Does It Really Matter?

**DOI:** 10.1021/jasms.2c00290

**Published:** 2023-02-06

**Authors:** Christian van der Linde, Milan Ončák, Ethan M. Cunningham, Wai Kit Tang, Chi-Kit Siu, Martin K. Beyer

**Affiliations:** † Institut für Ionenphysik und Angewandte Physik, Universität Innsbruck, Technikerstraße 25, 6020Innsbruck, Austria; ‡ Institute of Research Management and Services (IPPP), Research and Innovation Management Complex, University of Malaya, Kuala Lumpur50603, Malaysia; § Department of Chemistry, City University of Hong Kong, 83 Tat Chee Avenue, Kowloon Tong, Hong Kong SAR, PR China

## Abstract

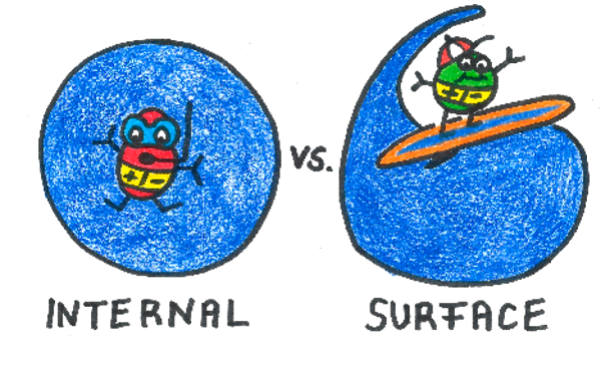

The precise location of an ion or electron, whether it is internally solvated or residing on the surface of a water cluster, remains an intriguing question. Subtle differences in the hydrogen bonding network may lead to a preference for one or the other. Here we discuss spectroscopic probes of the structure of gas-phase hydrated ions in combination with quantum chemistry, as well as H/D exchange as a means of structure elucidation. With the help of nanocalorimetry, we look for thermochemical signatures of surface vs internal solvation. Examples of strongly size-dependent reactivity are reviewed which illustrate the influence of surface vs internal solvation on unimolecular rearrangements of the cluster, as well as on the rate and product distribution of ion–molecule reactions.

## 1 Introduction

In bulk aqueous solution, ions are surrounded by water molecules, interacting via ion–dipole interactions and hydrogen bonding. Using the Born equation^1^ as the classic example of a continuum solvation model, the charge center interacts with a continuous dielectric medium, and quantitative predictions on the electrostatic contribution to the Gibbs energy of hydration can be made. X-ray photoelectron spectroscopy, however, revealed that bromide and iodide ions are enriched at the surface of aqueous solutions,^2^ indicating a preference for incomplete hydration for these relatively large anions. Size-selected ion–water clusters in the gas phase allow for a detailed investigation of the hydration environment of ions, molecule by molecule, and the impact of hydration on reactivity. Infrared spectroscopy combined with quantum chemistry reveals structural details,^3−5^ while ultraviolet/visible (UV/vis) and photoelectron spectroscopy provide information on electronic structure and photochemical reactivity.^6^ Black-body infrared radiative dissociation (BIRD) is ideal to study the relative stability of different cluster sizes.^7−10^ The reactivity of hydrated ions often depends on cluster size,^11^ a consequence of subtle changes in the reaction thermochemistry, as well as the accessibility of the ion by the neutral reactant, which originates from a gradual transition from surface to internal solvation with increasing cluster size.

Hydrated ions in the gas phase have been studied intensely since the 1970s, with seminal work such as the discovery of the magic (H_3_O)^+^(H_2_O)_20_ cluster by Searcy and Fenn^12^ or the determination of hydration enthalpies with high-pressure mass spectrometry by the groups of Kebarle^13−15^ and Castleman.^16−18^ Precise thermochemical information on the first solvation shell is obtained by guided ion beam (GIB) mass spectrometry in the Armentrout group,^19−23^ while BIRD has been less frequently used for the measurement of water binding energies.^24,25^ Ion spectroscopy in the infrared focuses on structural properties, with prominent contributions by Lee, Chang, and Niedner-Schatteburg,^26−28^ Duncan,^3^ Johnson,^29,30^ Okumura,^31,32^ Asmis,^33,34^ Dopfer,^35^ Ohashi,^36^ Williams,^37−39^ and Weber,^40^ among others. Photoelectron spectroscopy provides insight into electronic structure and dynamics, as studied in the groups of Bowen,^41^ Wang,^42^ and Neumark.^43,44^ Reactivity of ionic water clusters has received considerable attention, in particular as model systems for atmospheric chemistry, pursued, among others, by the groups of Fehsenfeld and Ferguson,^45^ Castleman,^46,47^ Okumura,^48^ and Bondybey and Niedner-Schatteburg.^49−51^

The question “how many molecules make a solution?”^52^ is intimately connected to the size dependence of the properties of hydrated ions. In particular, the transition from surface to internal solvation plays a major role. Size dependence, in particular, of chemical reactivity has many facets, with certain intracluster reactions occurring only in a narrow size regime. While the infrared absorption of a carbon dioxide radical anion converges to the bulk position with as few as 20 water molecules, the electronic absorption spectrum of a hydrated electron with as many as 200 water molecules still does not fully correspond to the spectrum measured in the bulk. Here, we discuss surface vs internal solvation of hydrated ions in terms of electronic and vibrational spectra, thermochemistry, and reactivity. We introduce selected examples from our laboratories and put them into perspective with the current literature. These examples show that the picture is quite complex. The transition from surface to internal solvation with increasing cluster size often proceeds rather gradually, but some ions simply remain on or near the surface, regardless of cluster size. Analysis of the origins and consequences of such behavior provides insight into solvation beyond classic continuum models.

## 2 Hydrated Ions in the Gas Phase

### 2.1 Structure

#### 2.1.1 vis/NIR Spectroscopy of the Hydrated Electron: (H_2_O)_*n*_^–^

**Figure 1:**
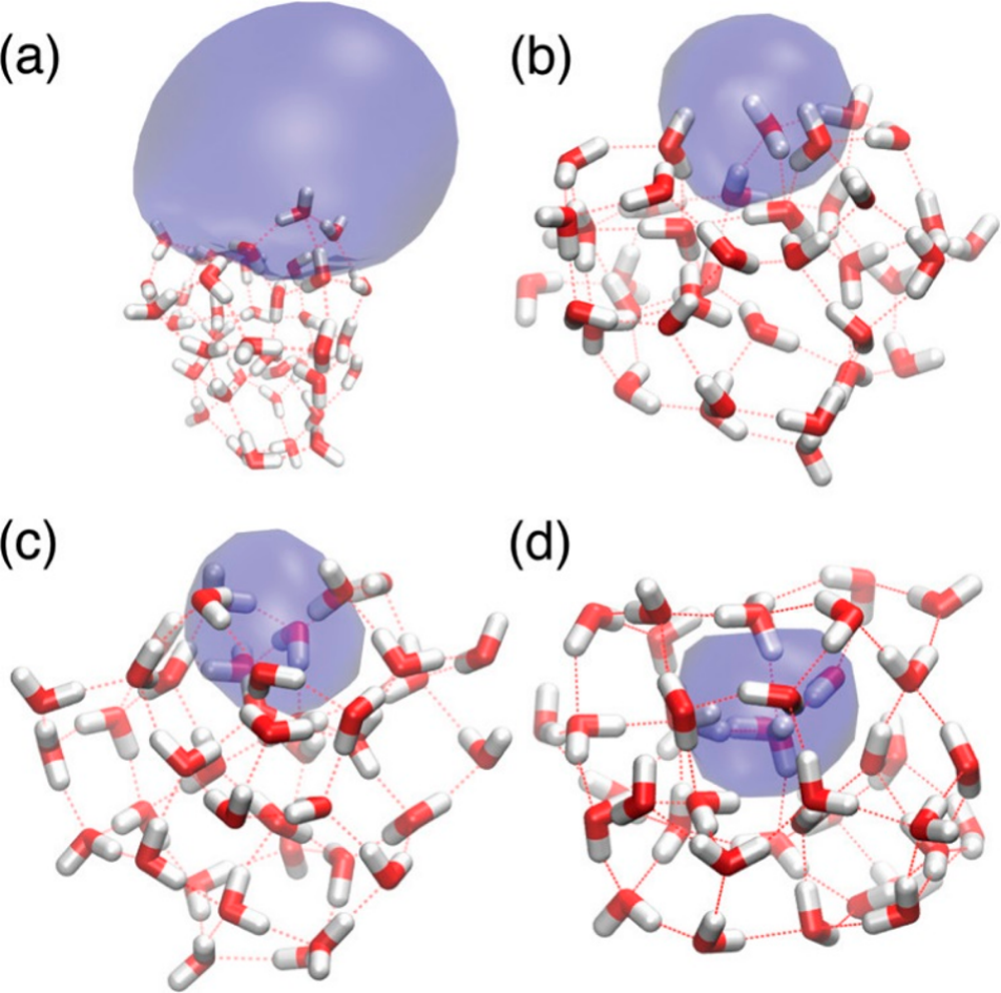
Four isomers of hydrated electron in (H_2_O)_40_^–^ along with an isosurface comprising 70% of the electron density: (a) a dipole-bound surface isomer, (b) a surface-bound isomer, (c) a partially embedded surface isomer, and (d) a cavity isomer. Oxygen atoms are red, hydrogen atoms white. Reproduced with permission from ref (54). Copyright 2011 American Chemical Society.

The hydrated electron is an intriguing example of a charge center interacting with a solvent environment.^43,53^Figure 1 shows four typical structural motifs of (H_2_O)_40_^–^, calculated by Jacobson and Herbert by mixed quantum/classical molecular dynamics.^54^ Water clusters show vast structural variability, and several arrangements are possible that lead to bound states of the electron. Water molecules may align to a high total dipole moment of the cluster, which sustains a dipole-bound state of the electron, see Figure 1a. Alternatively, the water molecules may rearrange in different ways to create a potential well for the electron, represented by the surface-bound, partially embedded and cavity bound isomers, also displayed in Figure 1.

**Figure 2:**
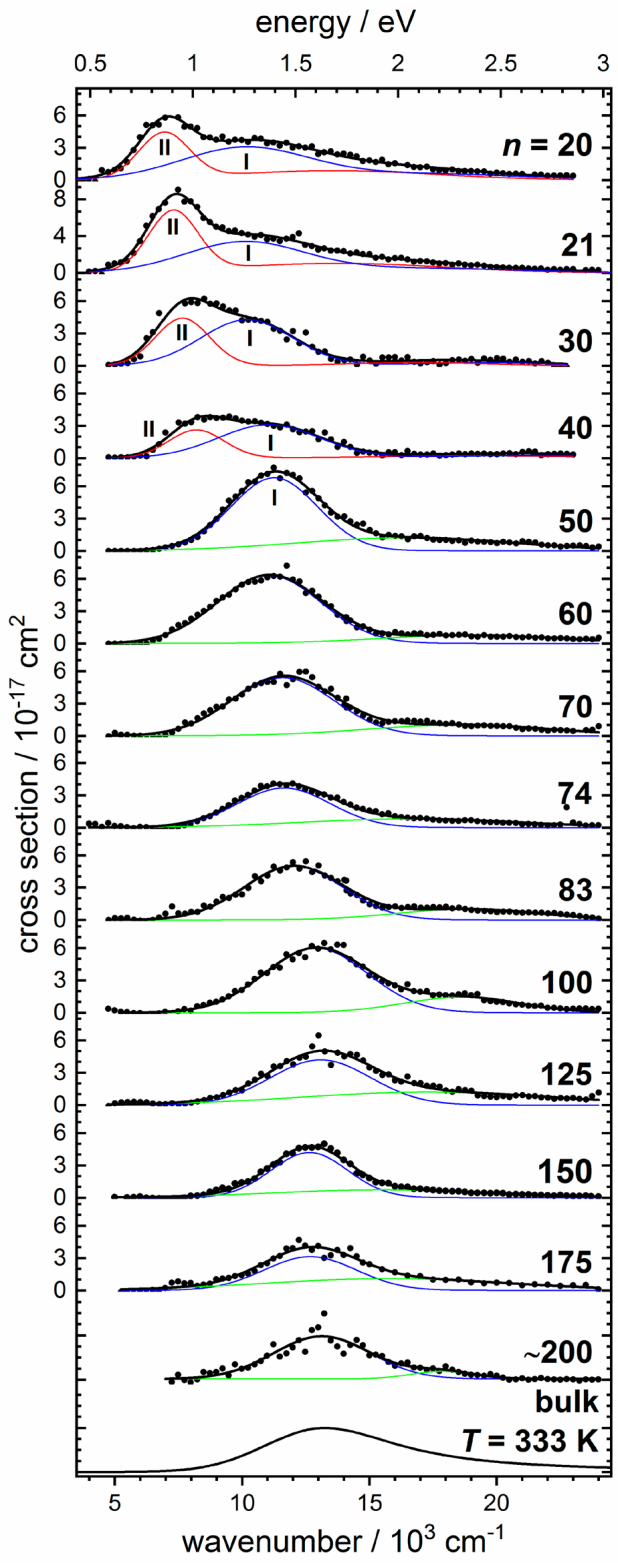
Absorption spectra of (H_2_O)_*n*_^–^ at a temperature of 80 K; the bulk spectrum of the hydrated electron at 333 K is taken from ref (57). Reproduced with permission from ref (55) under Creative Commons Attribution (CC-BY) license. Copyright 2019 The Authors.

Ayotte and Johnson showed that the electronic absorption spectra of (H_2_O)_*n*_^–^ are strongly size dependent.^6^ The photodissociation spectra (recorded with action spectroscopy) from our laboratory,^55^Figure 2, indicate the presence of at least two types of structural motifs for *n* = 20, 21, 30, and 40. In each spectrum, either the electron can detach or water molecules can evaporate. Electron detachment is monitored through depletion of ion signal, and water evaporation through detection of fragment ions. Both events were detected using mass spectrometry. Since excited states of the hydrated electron undergo ultrafast internal conversion,^43^ fluorescence does not play a role, and the photodissociation spectrum corresponds to the absorption spectrum. Type II is strongest at *n* = 20, 21, but steadily decreases for *n* = 30 and 40. For *n* ≥ 50, only type I is observed. Another intriguing aspect is the blue-shift of the band position of type I with increasing cluster size for *n* = 20–100. For larger clusters, however, the band position stays constant. Based on an analysis of the electron gyration radius, we identified type II as the surface-bound isomer, and type I as the partially embedded structure from Figure 1. In our experiments, the clusters are stored in a liquid-nitrogen cooled ion cyclotron resonance (ICR) cell at a temperature of 80 K under ultrahigh vacuum conditions, in an essentially collision-free environment. This temperature of 80 K is close to the solid-to-liquid phase transition reported by von Issendorff and co-workers,^56^ which suggests that sufficient internal energy is available for rearrangements of the clusters to their preferred structure. This indicates that the surface-bound and partially embedded isomers are very close in energy and are able to interconvert on the time scale of the ICR experiment, which is 100 ms to several seconds.

The structural motifs I and II discussed here are most likely different from the isomers I, II, and III observed in molecular beam experiments by Neumark and co-workers.^43,58−60^ In the cold conditions of the molecular beam and the short time scales of this experiment, at least two energetically higher-lying isomers, presumably surface-bound states of the electron, can be prepared. In the ICR experiment at 80 K, these clusters are heated by ambient blackbody radiation and either relax to the observed binding motifs, or detach the electron.

The absence of any shift in band position for 100 ≤ *n* ≤ 200 indicates that the binding potential well of the electron is unaffected by the increase in cluster size. Upon increasing the cluster size, water molecules are added to sites remote from the electron. The partially embedded type I hydrated electron corresponds closely to Jungwirth’s near-surface isomer for the bulk.^61^ However, a charge located near the surface and thus not fully solvated is against the intuition drawn from the dielectric continuum model. This discrepancy can be resolved by analyzing the energetic contributions, i.e., the binding energy of the electron in the potential well and the reorganization energy of the water network that creates the well, which in sum yield the adiabatic electron affinity. The binding energy of the electron in the potential well is equivalent to the vertical detachment energy (VDE), which was reported by Abel as 1.6 and 3.3 eV for surface- and interior-bound bulk hydrated electrons, respectively,^62^ while Signorell and co-workers place the VDE of the interior state at 3.7 eV,^63^ both based on liquid-jet experiments. The adiabatic electron affinity is given by Donald and Williams as 1.34 eV at an absolute scale from gas-phase cluster nanocalorimetry,^64^ while a bulk equilibrium measurement by Shiraishi et al.^65^ yields 1.78 eV when referenced to a proton hydration enthalpy of −1090 kJ mol^–1^. Coe argues that the proton hydration enthalpy is most likely significantly larger, estimating −1151 kJ mol^–1^ from extrapolation of cluster studies.^66^ This puts the solvent reorganization energy in the relatively broad range of 1.5–2.4 eV. A more exhaustive discussion of these values has been provided by Paesani and co-workers.^67^ Unfortunately, no general agreement has been reached on these fundamental thermochemical values.

**Figure 3:**
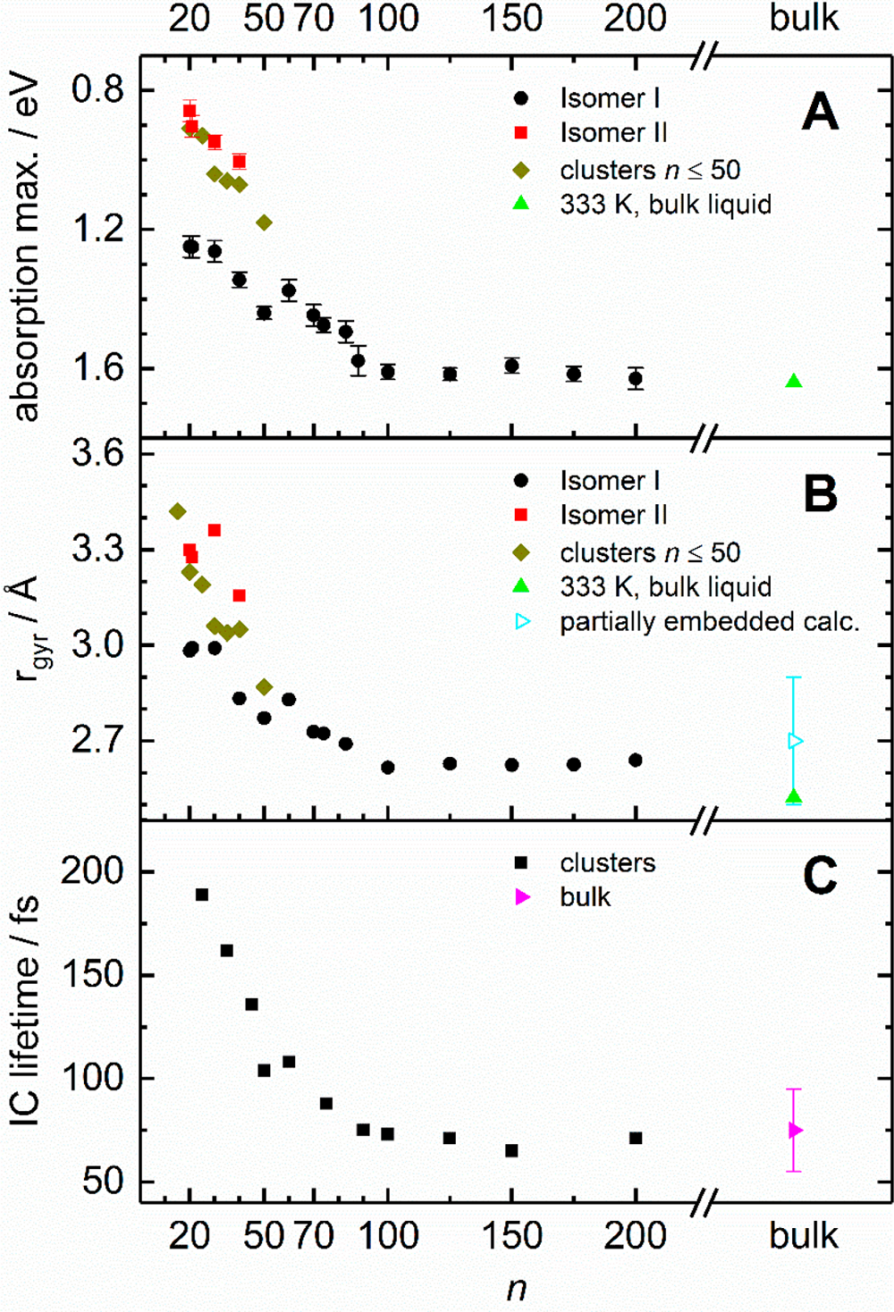
(A) Absorption maxima of isomers I and II shown in Figure 2 compared with previous data for clusters^6^ and hydrated electron.^57^ (B) Gyration radius of the electron for isomers from (A) along with calculations for partially embedded electron.^61^ (C) Internal conversion (IC) lifetime in the hydrated electron for (H_2_O)_*n*_^–^ clusters^68^ and bulk.^69^ Reproduced with permission from ref (55) under Creative Commons Attribution (CC-BY) license. Copyright 2019 The Authors.

Nevertheless, our electronic absorption spectroscopy study allows for some qualitative conclusions with respect to the surface vs interior isomers of the hydrated electron. The reported VDEs for these isomers differ considerably, but our study shows that the absorption spectrum appears to converge toward the bulk at *n* ≈ 200, Figures 2 and 3. Close convergence is also reached for internal conversion lifetime, which lies at around 75 fs. The electron gyration radius lies within 2.6–2.7 Å, slightly larger than the bulk value of 2.5 Å. Taken together, these observations strongly suggest that the partially embedded isomer resides in a potential well with a geometry very similar to the bulk hydrated electron. This implies that the differences in VDEs are almost entirely due to the different solvent reorganization energies, while the adiabatic binding energies of surface or interior states are almost the same. This requires a, probably fortuitous, compensation of the differences in VDEs and depths of the binding potential wells of surface and interior states.

Surface vs internal solvation thus makes a significant difference to the depth of the binding potential well and solvent reorganization energies of the hydrated electron, but has surprisingly little effect on the electronic states, radius of gyration, electronic absorption spectrum, and adiabatic electron affinity.

#### 2.1.2 Interfacial Effects on Ionization Energies

Similar conclusions have been reached by an in-depth analysis^70^ of liquid microjet photoelectron spectroscopy of salt solutions.^71−74^ It is intriguing to note that vertical ionization energies (VIEs) of hydrated inorganic anions, such as Cl^–^, NO_2_^–^, or CO_3_^2–^, measured by photoelectron spectroscopy, do not change significantly between surface or near surface solvation and the bulk, as recently shown by Paul and Herbert.^70^ In particular, soft anions may be present at the air/water or air/vacuum interface. Their calculations suggest that the first-shell hydration structure does not change significantly between surface or bulk solvation, which results in minor changes to the VIEs. In other words, it does not matter much for this experiment whether the ions are located at the surface or in the bulk. The authors conclude that the surface activity of soft anions arises from disruption of water–water hydrogen bonds, while the first solvation shell remains unaffected, i.e., the anions carry their first solvation shell to the surface.

#### 2.1.3 Electronic Spectroscopy of Hydrated Singly Charged Metal Ions

UV/vis spectra of V^+^(H_2_O)_*n*_, *n* = 1–8,^75^ and Al^+^(H_2_O)_*n*_, *n* = 1–8,^76^ exhibit a pronounced redshift with increasing coordination number of the metal center. For vanadium, the redshift stops at *n* = 4, which marks the completion of the preferred square-planar coordination of V^+^. For Al^+^(H_2_O), only one data point above the noise level at 225 nm could be recorded, since the tunable laser did not provide shorter wavelengths. For *n* = 2, a strong photodissociation signal was observed at 225 nm, leveling off toward 260 nm. No further redshift is observed beyond *n* = 2, suggesting that Al^+^ remains doubly coordinated for *n* ≤ 8. The reason for this behavior lies in the strongly polarizable 3s electron pair present in Al^+^, which weakens the interaction of incoming additional water molecules exceeding the first solvation shell.

For the electronic spectra of hydrated metal ions, each water molecule in the first solvation shell has a significant effect on the spectra, while adding water to the second or higher solvation shells does not lead to significant changes. As long as the metal center is not fully coordinated, one will consider it surface solvated, but a more precise description of the situation is the coordination number.

#### 2.1.4 Infrared Spectroscopy of the Hydrated Carbon Dioxide Radical Anion CO_2_^–^(H_2_O)_*n*_

Infrared (IR) spectroscopy is a standard tool in structure analysis. For hydrated ions in the gas phase, the O–H stretch is the most sensitive region to gather information on the hydrogen bonded network, as, e.g., applied by Williams and co-workers in the spectroscopy of SO_4_^2–^(H_2_O)_*n*_.^77^ In the present case, however, we focus on the IR absorptions of the CO_2_^–^ radical anion and how they change as a function of cluster size, *n*. In bulk aqueous solution, CO_2_^–^ exhibits a transient Raman band at 1298 cm^–1^, assigned to the symmetric C–O stretch.^78^ In the gas phase, the spectra are measured by infrared multiple photon dissociation (IRMPD),^79−81^ where the absorption of multiple photons causes evaporation of water molecules. The resulting mass change, as detected by mass spectrometry, thus provides the signature of absorption.

**Figure 4:**
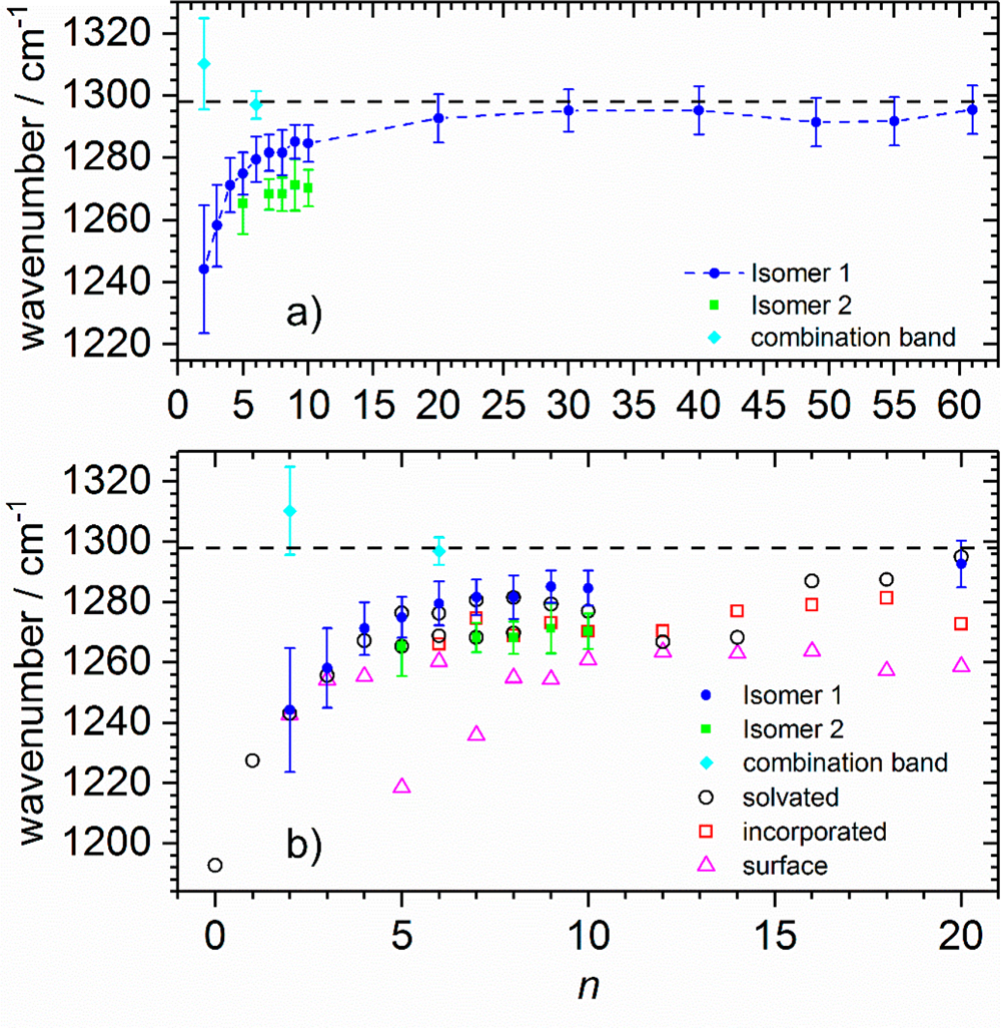
(a) Evolution of experimental band position and peak width indicated by error bars of the symmetric stretch ν_s_ with cluster size *n* for CO_2_^–^(H_2_O)_*n*_. The position of ν_s_ in bulk liquid water is indicated by a dashed line.^78^ (b) Calculated vibrational frequencies (open symbols) for ν_s_ at the B3LYP/6-311++G** level for *n* = 0–20, scaled by a factor of 0.977, compared with experiment (full symbols). Combination bands for *n* = 2, 6 are expected to arise from a combination of CO_2_^–^ bending and water libration. Reproduced and adapted with permission from ref (82) under Creative Commons Attribution (CC-BY) license. Copyright 2019 The Authors.

CO_2_^–^(H_2_O)_*n*_ ions were generated via laser vaporization of a Zn target, providing electrons, which were entrained in a gas pulse of helium, CO_2_, and water. The clusters were subsequently trapped in an ICR cell cooled to 80 K.^82^Figure 4a shows the band positions for selected clusters in the range of *n* = 2–61. The symmetric stretch starts at 1242 cm^–1^ for *n* = 2, whereby the signature for IR absorption is provided by electron detachment. IR absorption in this case is monitored by signal depletion, which leads to a larger error. Clusters *n* ≥ 3 all fragment by loss of individual water molecules. The band position blueshifts with growing hydration for *n* ≤ 20, and within error limits reaches the bulk value^78^ of 1298 cm^–1^ for 20 ≤ *n* ≤ 61, with a band position of 1296 cm^–1^ for the largest cluster size studied. The blue shift is attributed to the stabilization of the highest occupied molecular orbital (HOMO) by hydration, which leads to a higher force constant. However, this stabilization is basically fully accomplished with 20 water molecules, with further hydration leading only to minor shifts in the band position. Gauss fits to the spectra reveal small contributions of a second peak for some cluster sizes, which are assigned to combination bands or a second isomer.

**Figure 5:**
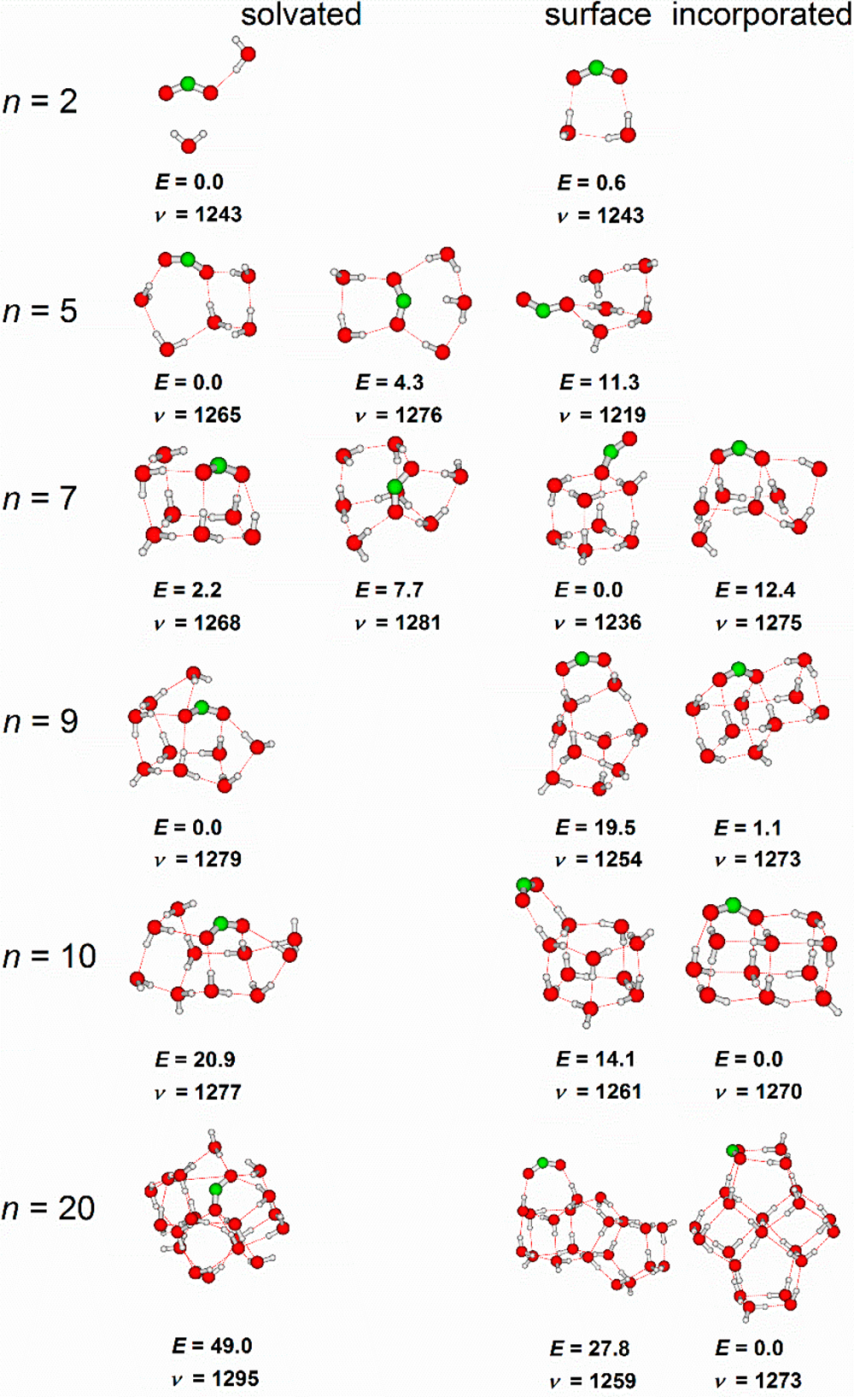
Selected isomers of CO_2_^–^(H_2_O)_*n*_ optimized at the B3LYP/6-311++G** level, along with the relative energy in kJ mol^–1^ and position of the symmetric stretch in CO_2_^–^ in cm^–1^ (scaled by 0.977). Reproduced with permission from ref (82) under Creative Commons Attribution (CC-BY) license. Copyright 2019 The Authors.

To understand the evolution of the symmetric stretch with cluster size, Figure 5 shows selected calculated low-energy structures of two types: solvated isomers and surface incorporated isomers. The solvated isomers feature a significant C···H interaction for *n* ≥ 7, with one O–H bond clearly pointing toward the carbon atom. The surface incorporated isomers are constructed by replacing one H_2_O molecule in a neutral cluster with CO_2_^–^ and reoptimizing the structure. In the studied size regime up to *n* = 20, each isomer class reflects the size dependence, but we cannot assign the experimental clusters to either of the two isomers (Figure 4b). However, the calculations show clearly that the bulk value of the symmetric C–O stretch is reached with CO_2_^–^ not fully surrounded by water molecules, but rather CO_2_^–^ residing on the cluster surface, even in the case of the solvated isomers featuring a pronounced C···H interaction. The bulk value is thus reached with a surface-solvated species. For the force constant of the CO_2_^–^ symmetric stretching mode, surface or internal solvation thus does not seem to matter.

#### 2.1.5 IR Spectroscopy of Hydrated Zn^+^ and Zn_2_^+^

**Figure 6:**
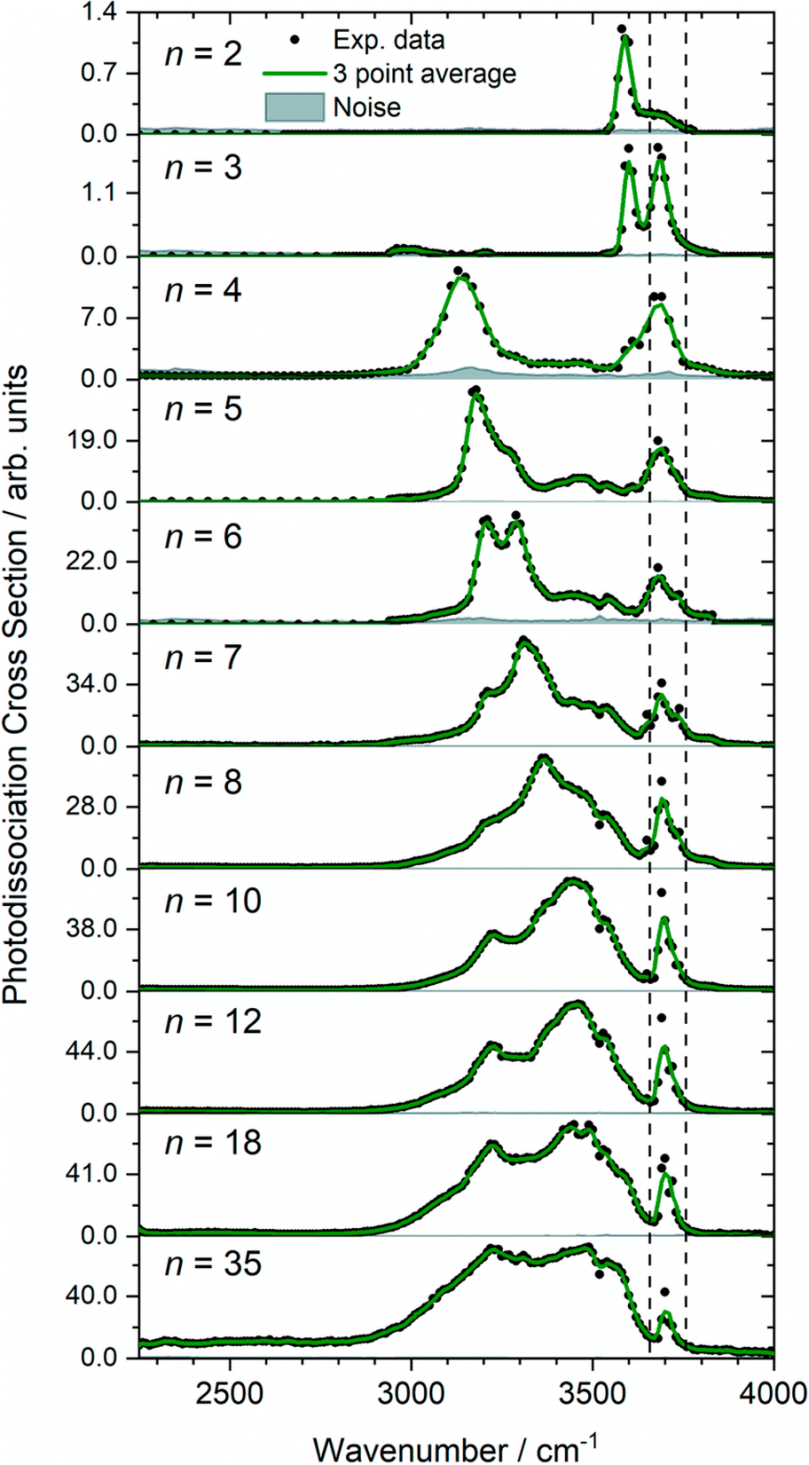
IRMPD spectra of Zn^+^(H_2_O)_*n*_ ions, where a one-photon process is assumed. The only observed dissociation event is water molecule loss. Symmetric and asymmetric stretching modes of isolated H_2_O are shown by dashed lines.^91^ Reproduced with permission from ref (87) under Creative Commons Attribution 3.0 Unported License. Copyright 2021 The Authors.

Reactivity studies on hydrated monovalent zinc ions with reactants like 1-iodopropane, acetonitrile, O_2_, HCl, or NO show that the Zn center can be readily oxidized.^11,83−86^ To gather information on the structural properties and solvation evolution of Zn^+^(H_2_O)_*n*_ complexes, IRMPD measurements were carried out on Zn cations hydrated with up to 35 water molecules, cooled to 80 K in the ICR cell.^87^Figure 6 shows the IRMPD spectra assuming a one-photon process for data analysis and laser power corrections, as explained in detail in the original work. The onset of the strong red-shifted band in the hydrogen bonding region (at 3130 cm^–1^) starts at four water molecules, concordant with the advent of the second solvation sphere, as previously observed by Duncan and co-workers.^88^ Consistent with density functional theory calculations, a coordination number of three is retained, even for the largest cluster size, whereby the 4s^1^ electron of Zn^+^ causes significant repulsion of incoming water ligands. The zinc dication, in contrast, was shown by Williams, Armentrout, and co-workers to reach a coordination number of five already with eight water molecules.^89^ Thus, the Zn^+^ ion resides on the surface of the water network, with the strongly distorted 4s orbital exposed, concordant with the reactivity studies with hydrophobic reagents O_2_ and IC_3_H_7_, which exhibited no clear size-dependence. Additionally, consistent with D_2_O exchange experiments,^90^ no evidence of a HZnOH^+^ motif was observed, which would present the infrared signature of a mobile proton at ca. 2800–3500 cm^–1^.

**Figure 7:**
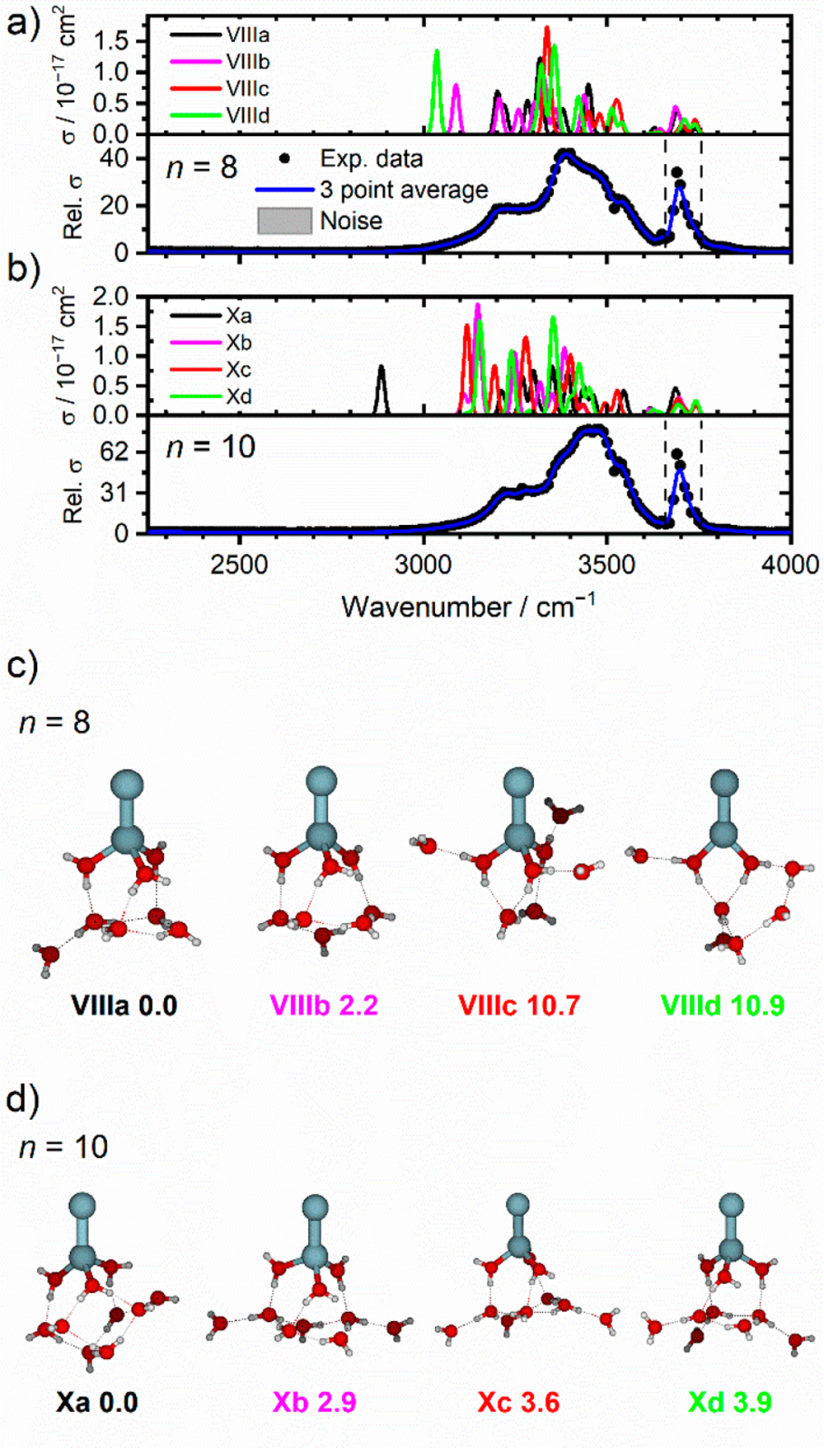
(a,b) Calculated IR spectra (above) and experimental IRMPD spectra (below) of Zn_2_^+^(H_2_O)_*n*_, *n* = 8, 10. Symmetric and asymmetric stretching modes of isolated H_2_O are shown by dashed lines.^91^ (c,d) Selected energetically low-lying isomers of Zn_2_^+^(H_2_O)_*n*_, *n* = 8, 10 as optimized at the B3LYP/aug-cc-pVDZ level, with relative energy given in kJ mol^−1^. Reproduced with permission from ref (92) under Creative Commons Attribution (CC-BY) license. Copyright 2021 The Authors.

Building on these findings, the solvation evolution and water binding interactions to the cationic zinc dimer, Zn_2_^+^, were investigated using IRMPD spectroscopy of Zn_2_^+^(H_2_O)_*n*_, *n* = 1–20.^92^ Together with simulated spectra of the thermodynamically most stable isomers generated using density functional theory, the observed spectra reveal evidence of asymmetric solvation, whereby water ligands bind exclusively to one of the Zn atoms in the dimer. By way of example, Figure 7 shows spectra and structures of low-lying isomers for *n* = 8, 10. For all cluster sizes investigated, the asymmetrically solvated isomers were calculated to lie lower in energy when compared to structural isomers where water molecules bind to both Zn atoms. Similar to the monatomic case, a coordination of three is retained for solvated Zn_2_^+^(H_2_O)_*n*_. Further evidence of asymmetric solvation is the loss of a neutral Zn atom for *n* = 3 and 4 water molecules. Calculated ligand binding energies show a pronounced decrease in Zn binding energy at *n* = 3, consistent with the observed IRMPD product.

Surface solvated metal cations are particularly counterintuitive. The two examples involving cationic zinc monomer and dimer illustrate nicely the reasons for such an asymmetric solvation, as discussed in detail before.^5^ Building the cluster molecule by molecule, each additional water molecule has the choice to either directly interact with the metal center via the oxygen atom, or integrate into the hydrogen bonded network, as long as a vacant coordination site is available. Surface solvation occurs when integration into the hydrogen bonded network is energetically preferred before the first hydration shell is filled.

#### 2.1.6 H/D Exchange As an Indirect Structural Probe

In bulk aqueous solution, autoprotolysis leads to rapid isotopic scrambling of H_2_O/D_2_O mixtures. For protonated water clusters, this behavior is indeed retained in the gas phase, as reported by Honma and Armentrout.^93^ However, we have shown that (H_2_O)_*n*_^–^, O_2_^–^(H_2_O)_*n*_, as well as M^+^(H_2_O)_*n*_, M = Cr, Fe, Co, Ni, Cu, Zn, do not undergo isotopic scrambling in reactions with D_2_O, i.e., H_2_O and D_2_O molecules stay intact.^90,94^ On the other hand, MgOH^+^(H_2_O)_*n*_ clusters, just like H^+^(H_2_O)_*n*_, rapidly exchange H and D isotopes, and the newly formed HDO molecules leaving the cluster serve as a mass spectrometric probe of the scrambling event.^94,95^ Similar observations were later made by Uggerud and co-workers.^96−100^ Johnson and co-workers ingeniously employ the effect that D_2_O stays intact in a neat H_2_O environment for elaborate pump–probe infrared spectroscopy studies.^101^

For Mn^+^(H_2_O)_*n*_ and Al^+^(H_2_O)_*n*_, however, we observed that the onset of H/D exchange depends sensitively on cluster size.^90,95^ H/D exchange is observed only for *n* ≈ 8–20 and *n* ≤ 38 for Mn^+^(H_2_O)_*n*_ and Al^+^(H_2_O)_*n*_, respectively. This indicates that a metal hydride-hydroxide structure is formed for these two metal ions, which does not lead to a mass change. In the case of aluminum, however, this hydride-hydroxide structure should persist also for larger clusters, since it is strongly thermochemically favored.^102,103^ One may speculate that integration of the HAlOH^+^ unit into a rigid hydrogen bonded network raises the barrier for proton transfer, which is a prerequisite for H/D exchange.

We have recently confirmed that the intracluster reaction of HAlOH^+^(H_2_O)_*n*−1_ to form molecular hydrogen and Al(OH)_2_^+^(H_2_O)_*n*−2_ proceeds via a concerted proton transfer reaction. It occurs with a minimum of about 12 water molecules, which coincides with the onset of hydrogen bonding to the hydride.^104^ In other words, molecular hydrogen is only formed when the HAlOH^+^ moiety is not surface solvated. Obviously, the transition from surface to internal solvation is crucial for this particular intracluster reaction.

### 2.2 Thermochemistry and Nanocalorimetry

The relation between gas-phase thermochemistry of clusters and solution phase hydration enthalpies has been studied extensively since the early 1970s, starting with the pioneering studies of the groups of Kebarle and Castleman employing high-pressure mass spectrometry.^18,105,106^ Based on the Thomson equation, single-ion heats of solvation can be obtained by extrapolation of gas-phase enthalpies for successive clustering reactions.^18^ Lee, Keesee, and Castleman also noted that beyond about 10 water molecules, the binding energy of additional water molecules is not significantly affected by the nature of the hydrated ion.^18^ For the hydrated electron, however, with its significant spatial requirements, this threshold may be higher. One may expect that beyond 50 water molecules, the thermochemistry of gas-phase hydrated ions closely resembles bulk aqueous solution. Williams and co-workers have employed this idea to perform electrochemistry in the gas phase, allowing free electrons to recombine with hydrated multiply charged cations trapped in an FT-ICR mass spectrometer.^64^ In these experiments, the cluster is treated as a nanocalorimeter. The heat of the reaction is released into the cluster, causing the evaporation of water molecules. By counting the number of evaporated water molecules directly via mass spectrometry, the reaction energy can be obtained with high accuracy.

#### 2.2.1 Benchmark Reaction: (H_2_O)_*n*_^–^ with SF_6_

**Figure 8:**
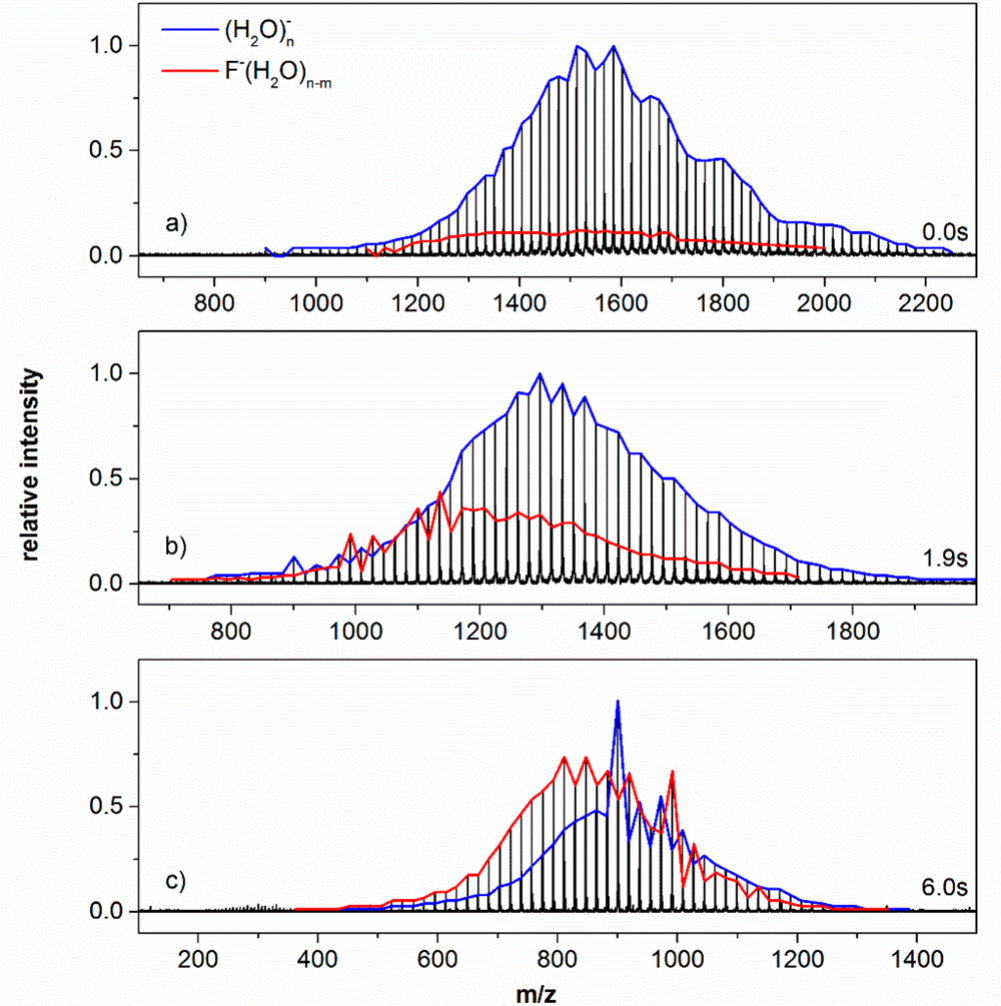
Mass spectra of the (H_2_O)_*n*_^–^ reaction with SF_6_ after varying time delay at *T* = 170 K. At *n* ≈ 50–60, (900–1080 *m*/*z*), (H_2_O)_50_^–^ (900 *m*/*z*), and F^–^(H_2_O)_54_ (991 *m*/*z*) magic numbers are observed. Reproduced and adapted with permission from ref (108). Copyright 2015 American Chemical Society.

We have developed a variant of nanocalorimetry that works with a broad cluster size distribution.^107^ To benchmark the method and compare with solution phase thermochemistry, we studied the reaction of hydrated electrons with sulfur hexafluoride, reaction 1, to form hydrated fluoride and an SF_5_ radical:^108^
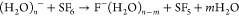
1Figure 8 shows mass spectra of the reaction after 0.0, 1.9, and 6.0 s reaction delay, recorded at a temperature of 170 K. At this temperature, BIRD is already substantially suppressed, while SF_6_ is not sticking to the cold surfaces, and the reactant pressure can be controlled. SF_6_ is taken up by the (H_2_O)_*n*_^–^ clusters and reacts to form F^–^(H_2_O)_*n*−*m*_, releasing SF_5_ together with *m*H_2_O molecules. For each individual event, *m* is an integer value that depends not only on the reaction energy but also on the internal energy of the cluster before the SF_6_ uptake. Since the internal energy distribution is rather broad, two or three different values of *m* are possible.

**Figure 9:**
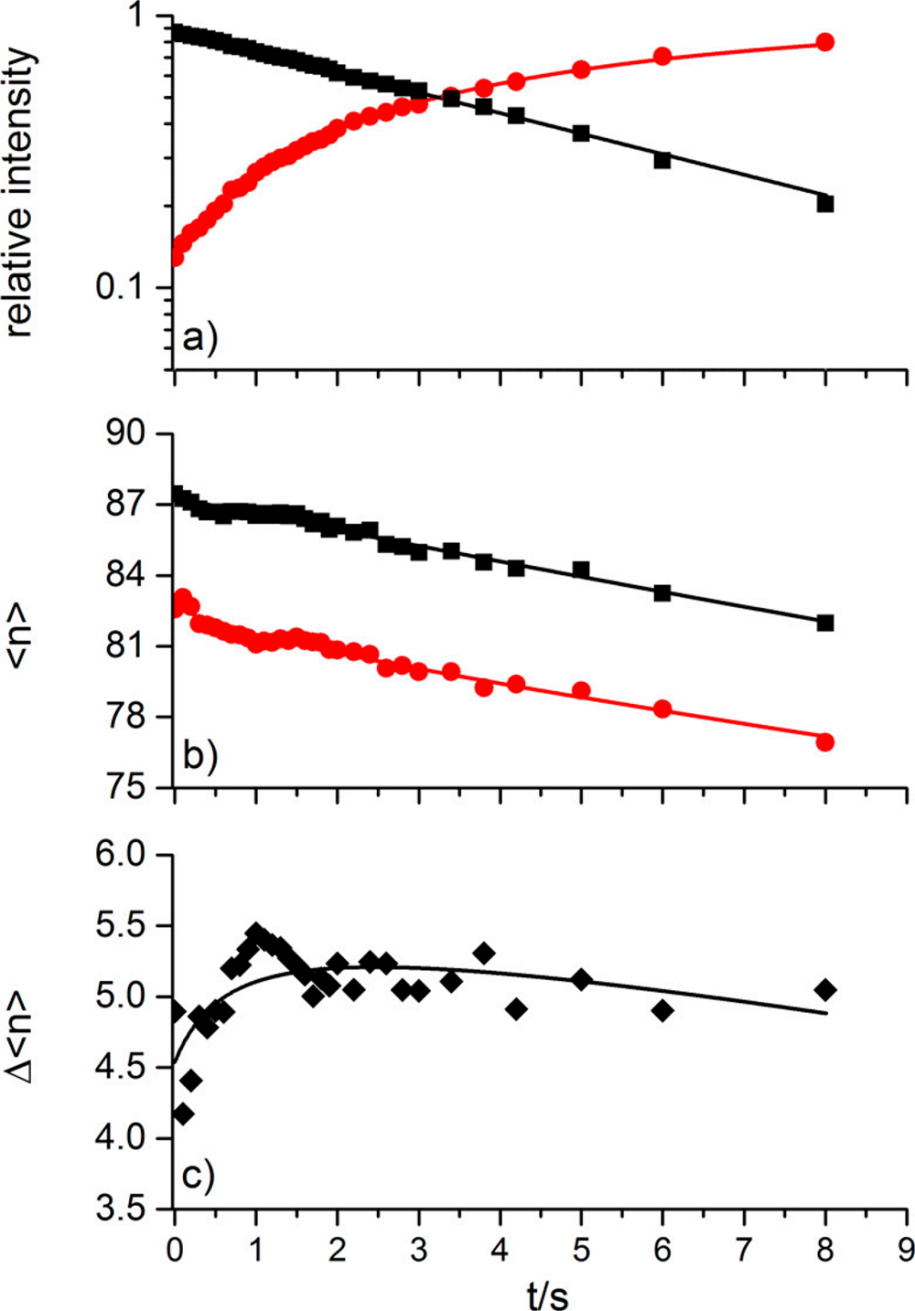
(a) Kinetics for reaction of (H_2_O)_*n*_^–^ (black) with SF_6_ to form F^–^(H_2_O)_*n*−Δ*n*_ (red) at *T* = 170 K along with a pseudo-first-order fit (line). (b,c) Nanocalorimetric analysis of the reaction showing average size ⟨*n*⟩ of reactant and product clusters and their difference Δ⟨*n*⟩. Reproduced with permission from ref (108). Copyright 2015 American Chemical Society.

Since the experiment is performed without mass selection, we obtain *m* from the analysis of the average cluster size ⟨*n*⟩ of reactant and product cluster distribution as a function of time. Figure 9a shows the aggregated intensities, which exhibit pseudo-first-order behavior. The rate coefficient obtained from this fit is used in the analysis of the average cluster sizes, which are described by a set of differential eqs 2 and 3:
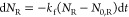
2
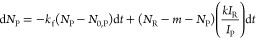
3where *N*_R_, *N*_P_ are the average cluster sizes of reactant and product, respectively, and *I*_R_, *I*_P_ the corresponding total intensities. The unimolecular rate coefficient, *k*_*f*_, together with the offsets *N*_0,R_, *N*_0,P_ describe the loss of water molecules due to BIRD.^9^ The pseudo-first-order rate coefficient, *k*, is related to the bimolecular reaction 1 and is obtained from the fit in Figure 9a. The fit of *N*_R_, *N*_P_ shown in Figure 9b yields the average number of water molecules *m* that evaporate due to the reaction. To increase the stability of the fit, the difference between reactant and product cluster sizes is used in addition, Figure 9c. We performed the experiment 8 times at different temperatures. Averaging the results yields a value *m* = 5.4 ± 0.4 evaporating water molecules, which is equivalent to a reaction enthalpy of Δ*H*_298 K_(1) = −234 ± 24 kJ mol^–1^, using previously established water binding energies to large water clusters.^56,109^

We then combined this result with other thermochemistry values for bulk aqueous solution in a thermochemical cycle to derive the F_5_S–F bond dissociation energy, see Table 1. The resulting value of Δ*H*_298 K_(F_5_S–F) = 455 ± 24 kJ mol^–1^ is within error limits of all high-level quantum chemical calculations in the literature.^112,113^ It is slightly above the presumably best experimental value of Δ*H*_298 K_(F_5_S–F) = 420 ± 10 kJ mol^–1^, suggested by Tsang and Herron after critical evaluation of the literature.^114^ This indicates that the concept of nanocalorimetry works very well and underlines again that gas-phase cluster thermochemistry closely reflects the situation in bulk aqueous solution. With respect to the error bars, one should consider that the strength of a water hydrogen bond is typically 20 kJ mol^–1^. Given the complexity of hydrogen bonded networks, in particular the large difference in solvent reorganization energy between surface-solvated ions or electrons in the gas phase and their bulk counterparts, an agreement of thermochemical values in the range of a single hydrogen bond is astonishing. Nanocalorimetry cannot compete with the accuracy of guided ion beam experiments from the Armentrout group.^115^ However, in special cases it can provide thermochemical values which are not accessible by any other method.

**Table 1:** Thermochemical Cycle for the F_5_S–F Bond Dissociation Energy^a^

Reaction	Δ*H*_298 K_ (kJ mol^–1^)	Ref
H^+^_(g)_ + e^–^_(g)_ →H^+^_(aq)_ + e^–^_(aq)_	–1261.9 ± 3.8	(65)
H^+^_(aq)_ + F^–^_(aq)_ →HF_(g)_	+61.5 ± 0.8	(110)
HF_(g)_ →H^•^_(g)_ + F^•^_(g)_	+570.7 ± 0.8	(111)
H^•^_(g)_ →H^+^_(g)_ + e^–^_(g)_	+1318.4 ± 0.0	(111)
SF_6(g)_ + e^–^_(aq)_ →SF_5_^•^_(g)_ + F^–^_(aq)_	–234 ± 24	(108)
SF_6(g)_ →SF_5_^•^_(g)_ + F_(g)_	+455 ± 24	sum of all above

a Reproduced and adapted with permission from ref (108). Copyright 2015 American Chemical Society.

Agreement within error limits was also reached for the F_2_ClC–Cl bond dissociation energy in CF_2_Cl_2_ derived in a similar way,^116^ Δ*H*_298 K_(F_2_ClC–Cl) = 355 ± 41 kJ mol^–1^ for nanocalorimetry comparing with a bond dissociation energy of 346.0 ± 13.4 kJ mol^–1^ derived from standard enthalpies of formation.^117,118^

Combining gas-phase nanocalorimetry of hydrated ions with solution phase thermochemistry, as exemplified in Table 1, relies on the assumption that potential differences of the hydration enthalpies between surface and internal solvation either compensate each other, or are negligible. In section 2.1.1, we have argued that for the hydrated electron, the adiabatic binding energy does not differ much between surface vs. internal solvation. The agreement within error limits reached in Table 1 for the F_5_S–F bond dissociation energy suggests that this also largely holds for F^–^, for which older calculations predict internal solvation for *n* = 15,^119^ while more recent works report surface solvation for *n* ≤ 10.^120,121^ However, the situation may be different for other ions, e.g., larger molecular ions with a delocalized charge distribution.

#### 2.2.2 One-Electron Reduction of Organic Molecules

Birch reduction of aromatic compounds is performed with the help of electrons solvated in liquid ammonia.^122^ We observed a one-electron reduction, which corresponds to the first step of Birch reduction, in gas-phase reactions of hydrated electrons with acetonitrile,^123^ chlorobenzene,^124^ and with all di- and trifluorobenzene isomers.^125^ Although abstraction of the halogen atom to form F^–^(H_2_O)_*n*−*m*_ is energetically favored by more than 100 kJ mol^–1^, the mildly exothermic reduction product OH^–^(H_2_O)_*n*−*m*−1_ is observed for all isomers, and in most cases is the dominant product. In that study, thermochemical values from gas-phase nanocalorimetry were compared with a combination of literature thermochemistry for bulk aqueous solution and quantum chemistry on the G3 level. For the one-electron reduction, nanocalorimetry yields Δ*E*_nc_ = −22 ± 13 kJ mol^–1^, while fluorine abstraction results in Δ*E*_nc_ = −144 ± 32 kJ mol^–1^. Both values agree within error limits with the literature/G3 result of Δ*H*_aq_ = −23 kJ mol^–1^ and Δ*H*_aq_ = −126 kJ mol^–1^, respectively. Again, there is no sign of a significant influence of surface solvation in the cluster vs internal solvation in the bulk.

#### 2.2.3 Hydration Enthalpies of CO_2_^–^ and O_2_^–^

Having tested the error margins of gas-phase nanocalorimetry for condensed phase thermochemistry, we used the uptake of CO_2_ and O_2_ by (H_2_O)_*n*_^–^ to estimate the solution-phase hydration enthalpy of their radical anions.^126^Table 2 shows the results obtained again by combining gas-phase nanocalorimetry with solution phase thermochemistry, compared with earlier studies from different groups. We obtained Δ*H*_hyd_(CO_2_^–^) = −334 ± 44 kJ mol^–1^ and Δ*H*_hyd_(O_2_^–^) = −404 ± 28 kJ mol^–1^, which compare favorably with earlier results from Posey et al.,^127^ while Arnold et al.^128^ report a smaller number of evaporating water molecules.

**Table 2:** Reaction Rates, *k*_abs_, Number of Evaporated Water Molecules, Δ*N*_vap_, and Reaction and Hydration Enthalpy, Δ_r_*H* and Δ_hyd_*H*, respectively, for Three Reactions of (H_2_O)_*n*_^–^ with O_2_ and CO_2_^a^

Reaction	Source	*k*_abs_ (cm^3^ s^–1^), *T* = 298 K	Δ*N*_vap_	Δ_r_*H* (kJ mol^–1^)	Δ_hyd_*H* (kJ mol^–1^)
CO_2_ + (H_2_O)_*n*_^–^	Akhgarnusch et al.^b^	9.8 × 10^–10^	2.46 ± 0.75	–105 ± 39	–334 ± 44^c^
	Höckendorf et al.^d^	1.0 × 10^–9^	1.0 ± 0.2	–39 ± 9	–268 ± 27
	Arnold et al.^e^	7.6 × 10^–10^	1.3	–	–
	Posey et al.^f^	–	3	–105.2^g^	–333.8^h^
	Balaj et al.^i^	–	2–3	–	–
O_2_ + (H_2_O)_*n*_^–^	Akhgarnusch et al.^b^	1.4 × 10^–10^	6.40 ± 0.45	–276 ± 28	–404 ± 28^c^
	Höckendorf et al.^d^	5.4 × 10^–10^	5.8 ± 0.2	–247 ± 20	–375 ± 30
	Arnold et al.^e^	2.5 × 10^–10^	5.0	–	–
	Posey et al.^f^	–	7	–317^g^	–445.8^h^
	Balaj et al.^i^	–	5–6	–	–
O_2_ + CO_2_^–^(H_2_O)_*n*_	Akhgarnusch et al.^b^	3.7 × 10^–11^	3.40 ± 0.63	–146 ± 29	–
	Höckendorf et al.^d^	4.1 × 10^–11^	3.5 ± 0.2	–149 ± 14	–
	Balaj et al.^i^	–	3–4	–	–

a Reproduced with permission from ref (126) under Creative Commons Attribution 3.0 Unported Licence. Copyright 2016 The Authors.

b Ref (126).

c Referenced to Δ_hyd_*H*(H^+^) = −1090 kJ mol^–1^.

d Ref (107).

e Ref (128).

f Ref (127).

g Estimated reaction enthalpy from ref (127) combined with the electron hydration enthalpy from ref (65) referenced to Δ_hyd_*H*(H^+^) = −1090 kJ mol^–1^.

h Estimated in ref (127) based on data from refs (129) and (18).

i Ref (130).

When we extrapolate gas-phase thermochemistry to bulk values, the underlying assumption is that the differences between gas-phase clusters and the bulk are similar for reactants and products. When we place reactant and product clusters into the bulk in a *Gedankenexperiment*, this should then require the same solvent reorganization energy. This idea will work best in cases where cluster structures are quite similar, in particular when both ions are internally solvated, with several layers of water molecules. In the case of surface or near-surface solvation, e.g., CO_2_^–^(H_2_O)_*n*_, O_2_^–^(H_2_O)_*n*_, or (H_2_O)_*n*_^–^, however, the integration of the cluster into the bulk network of hydrogen bonds will exhibit subtle differences. For this reason, one cannot expect that gas-phase nanocalorimetry represents bulk thermochemistry better than within the energy of a single hydrogen bond.

### 2.3 Gas-Phase Reactivity of Hydrated Ions

Since a neutral reactant molecule must be able to get in touch with the reactive center in the cluster, surface vs internal solvation may have a significant impact on the rate of ion–molecule reactions. The rate may even become strongly size dependent, if there is a transition from surface to internal solvation. Reactivity studies may thus provide important information on structural properties of clusters, in particular with respect to surface vs internal solvation.

#### 2.3.1 Hydrated Electrons

Gas-phase hydrated electrons react efficiently with molecules that form strong hydrogen bonds. While HCl dissociates in the cluster, releasing atomic hydrogen, reaction 4,^131^ dynamics steer the reaction of HNO_3_ toward the formation of OH^–^ and release of NO_2_, reaction 5.^132^ For these reactions, it is probably irrelevant whether the electron is located at the surface, partially embedded or in a cavity.
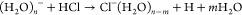
4
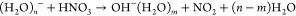
5The uptake of CO_2_ by (H_2_O)_*n*_^–^ proceeds with 30–70% efficiency, depending on the model employed for the calculation of the collision rate.^126,133^ On the other hand, uptake of O_2_ is considerably less efficient, with 6–11% at room temperature. Since clusters with 50–100 water molecules cannot be treated as a point charge, collision models must be used that take the geometric extension of the clusters into account, such as the hard-sphere average dipole orientation theory (HSA) or the surface-charge capture (SCC) models.^133^ It was recognized by Arnold et al. that the triplet ground state of O_2_ reduces the rate of the reaction, since the spins of the hydrated electron and the O_2_ molecule must be aligned antiparallel to afford a doublet product state.^128^ However, this explains the reduced efficiency only in part. Since the nonpolar molecules CO_2_ and O_2_ do not interact strongly with the hydrogen bonded network of the cluster, one may speculate that formation of the radical anion takes place only if the reactant molecules collide with the cluster in the vicinity of the electron.

Like with CO_2_ and O_2_, only one reactant molecule is taken up if a stable hydrated radical anion is formed, and further reactant molecules interact only weakly with the water cluster and the new core ion, as is the case for acetone.^134^ Nitromethane, acetaldehyde, and benzaldehyde also form radical anions, but additional reactant molecules are taken up.^135^ However, also more complex reaction pathways are possible, like the oligomerization of acrylic acid, the elimination of methanol from two molecules of methyl acrylate, or the delayed dissociation of vinyl acetate in the water cluster after reacting with the hydrated electron.^136^ Dissociative electron attachment in gas-phase hydrated electrons is observed with CH_3_SH and, to a small extent, CH_3_SSCH_3_.^137^ All these reactions start with the recombination of the neutral reactant with the hydrated electron, forming a solvent-stabilized radical anion. The reactions are very efficient if the reactant integrates into the hydrogen bonded network of the water cluster, and the binding motif of the hydrated electron does not really matter in this case.

#### 2.3.2 Reactions of CO_2_^–^(H_2_O)_*n*_

Consistent with the higher exothermicity of O_2_ vs CO_2_ uptake by hydrated electrons, CO_2_^–^(H_2_O)_*n*_ clusters react with O_2_ in a core exchange reaction. The CO_2_^–^(H_2_O)_*n*_ clusters are transformed into O_2_^–^(H_2_O)_*n*_ upon collision with molecular oxygen, reaction 6.^107,126,130^
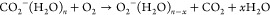
6

Nanocalorimetric analysis^126^ yields a reaction energy of Δ*E*_nc_(6) = −146 ± 29 kJ mol^–1^. This is within error limits consistent with the nanocalorimetric analysis of the O_2_ and CO_2_ uptake by hydrated electrons, see Table 2. The earlier reported nonergodic component in the core-exchange reaction^107^ originated from an error in the enthalpy of CO_2_ uptake by the hydrated electron.^126^ Interestingly, the core exchange reaction 6 proceeds an order of magnitude slower than the O_2_ uptake by hydrated electrons, Table 2. This is explained by the formation of a CO_4_^–^ intermediate, as predicted by Weber^138^ and confirmed by our work.^126^ The oxygen molecule must be able to approach the carbon dioxide radical anion for CO_4_^–^ formation, which is afforded by its position on the surface of the cluster.

With the unpaired electron localized at the carbon atom, CO_2_^–^(H_2_O)_*n*_ clusters may be expected to react with unsaturated hydrocarbons via radical addition.^139^ While ethylene and vinyl acetate are unreactive, methyl acrylate,^140^ allyl alcohol^141^ as well as 3-butyn-1-ol^142^ exhibit the expected reactivity, exemplified in reaction 7 for methyl acrylate.
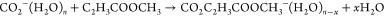
7

After long reaction delays of 35 s, the clusters form CO_2_C_2_H_3_COOCH_3_^–^(H_2_O) due to water loss via BIRD. The next steps in the blackbody radiation activated decomposition are loss of CO_2_ followed by electron detachment from C_2_H_3_COOCH_3_^–^. The reaction proceeds efficiently, with a rate coefficient of *k*_abs_ = 1.6 × 10^–9^ cm^3^ s^–1^. Nanocalorimetry reveals that uptake of one methyl acrylate molecule leads to evaporation of 2.2 ± 0.5 water molecules, equivalent to a reaction enthalpy of Δ*E*_nc_(7) = −95 ± 22 kJ mol^–1^.^107^ The fact that the uptake stops after one methyl acrylate molecule, along with the exothermicity of the reaction, indicates that a covalent bond is formed between CO_2_^–^ and methyl acrylate. Quantum chemical calculations are quantitatively consistent with this assumption and experimental findings.^140^

Textbook radical chemistry is also observed in reactions of CO_2_^–^(H_2_O)_*n*_ with methyl mercaptan and dimethyl disulfide.^137,143^ With methyl mercaptan, a hydrogen atom transfer takes place.^143^ In the case of dimethyl disulfide, the radical attack weakens the S–S bond, leading ultimately to its cleavage, with formation of CH_3_SCO_2_^–^(H_2_O)_*n*_. In both cases, a CH_3_S radical is released.^137^

Apart from radical chemistry, we also probed acid–base reactions of CO_2_^–^(H_2_O)_*n*_.^144^ While HOCO is released only to a small extent in reactions with HCl, abundant formation of NO_3_^–^(HNO_3_)_1,2_ indicates that HOCO is formed in the course of HNO_3_ uptake and BIRD.^144^

Neutral molecular oxygen does not integrate into the hydrogen bonded network of a water cluster and thus needs direct access to CO_2_^–^ in order to form CO_4_^–^, thus the core exchange reaction requires surface solvated CO_2_^–^. All other reactants, however, participate in the hydrogen bonded network for a sufficiently long period of time to reach the CO_2_^–^ reactive center, evidenced by the high rate coefficients of these reactions. In these cases, reactivity does not rely on surface solvated CO_2_^–^.

#### 2.3.3 Reactions of [Mg(H_2_O)_*n*_]^+^

As suggested by Fuke and co-workers,^145,146^ further supported through BIRD and reactivity experiments by Niedner-Schatteburg, Bondybey, and co-workers,^147,148^ calculated with DFT methods by Reinhard and Niedner-Schatteburg^149^ as well as Siu and Liu,^150,151^ and experimentally confirmed by electronic photodissociation spectroscopy in our group,^152^ [Mg(H_2_O)_*n*_]^+^ with *n* > 15 consists of hydrated Mg^2+^ and a hydrated electron. The evolution of the hydrated electron is nicely reflected in photodissociation spectra of Mg^+^(H_2_O)_*n*_, *n* = 1–5, which exhibit a strong redshift with increasing coordination number.^146,153^ This redshift originates from the increased polarization of the Mg^+^ 3s electron upon hydration, which ultimately leads to the complete displacement of the electron density from the metal center. The driving force for this behavior is the strong interaction^154^ of Mg^2+^ with up to six solvating water molecules.

The first experiment to detect the presence of the hydrated electron was the reactivity of [Mg(H_2_O)_*n*_]^+^ with HCl.^148^ Similar to (H_2_O)_*n*_^–^ discussed above, uptake of the first HCl molecule leads to elimination of a hydrogen atom, reaction 8, supporting the idea of intracluster charge separation.
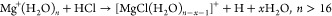
8In a similar fashion, uptake of O_2_ and CO_2_^155^ as well as the CO_2_/O_2_ exchange reaction^156^ proceed qualitatively in the same way as with hydrated electrons. Mg^+^(H_2_O)_*n*_, *n* ≈ 20–60, clusters take up one reactant molecule in the reaction with O_2_ and CO_2_, reactions 9 and 10.
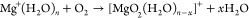
9
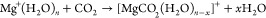
10
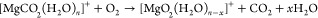
11The observed reactivity resembles the reactions of the hydrated electron (H_2_O)_*n*_^–^ with O_2_ and CO_2_.^130^ The reaction rate coefficients for CO_2_ uptake (reaction 9) given in Table 3, however, are significantly smaller than for the hydrated electron.^107^ Also, O_2_ uptake (reaction 10) and the CO_2_/O_2_ exchange reaction 11 proceed with a slightly reduced rate coefficient compared to the hydrated electron.^156^ Calculations employing density functional theory (DFT) on the solvation structure of the Mg^+^(H_2_O)_16_ predict that the clusters have a hexa-coordinated Mg^2+^ center ion and a remotely solvated electron, Mg^2+^(e^–^)(H_2_O)_16_. Hexacoordination of Mg^2+^ was also reported in room temperature aqueous solution by Havenith and co-workers,^157^ but the influence of the metal center in the far-infrared spectrum was confined to the first solvation shell. The calculations show that the ion–molecule reactions between Mg^+^(H_2_O)_16_ and O_2_ or CO_2_ are highly exothermic. In a neat water cluster, all water molecules can rearrange to accommodate the electron and at the same time maximize hydrogen bonding. With the Mg^2+^ ion nearby, however, the hydrogen bonding network also has to interface to the hexahydrated dication. Uptake of either O_2_ or CO_2_, as well as the exchange of CO_2_ against O_2_, with formation of hydrated O_2_^–^ or CO_2_^–^, respectively, requires extensive rearrangement of the hydrogen bonded network. Higher barriers due to the presence of Mg^2+^ for the uptake of these non-hydrogen bonding molecules help explain the reduced rates.

**Table 3:** Room Temperature Rate Coefficients, *k*_abs_ (10^–11^ cm^3^ s^–1^), of the Reactions of Mg^+^(H_2_O)_*n*_ and (H_2_O)_*n*_^–^ with CO_2_ and O_2_ and the CO_2_/O_2_ Exchange Reactions 6 and 11

	Mg^+^(H_2_O)_*n*_	(H_2_O)_*n*_^–^
CO_2_	1.9^a^	92^c^
O_2_	9.1^a^	13^d^
CO_2_/O_2_ exchange	2.4^b^	4.2^e^

a Ref (155).

b Ref (156), *n* ≈ 55.

c Ref (126), *n* = 40–92.

d Ref (126), *n* = 44–96.

e Ref (126), *n* = 41–114.

Interestingly, H atom formation in [Mg(H_2_O)_*n*_]^+^ under the influence of room temperature blackbody radiation is quenched upon uptake of either O_2_ or CO_2_. This shows that the hydrated electron is required for H atom elimination, and its scavenging by O_2_ or CO_2_ shuts off this reaction channel.

The hydrated electron is also reflected in the reaction of Mg^+^(H_2_O)_*n*_ (*n* ≈ 20–60) with CH_3_CN, which results in magnesium hydroxide MgOH^+^(H_2_O)_*n*−1_ and a neutral CH_3_CHN or CH_3_CNH radical, reaction 12.^158,159^
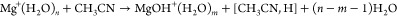
12
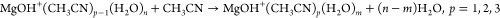
13Again, the observed reactivity is similar to the reaction of hydrated electrons (H_2_O)_*n*_^–^ with CH_3_CN.^123^ Up to three more CH_3_CN molecules are taken up by the MgOH^+^(H_2_O)_*m*_ clusters, and MgOH^+^(CH_3_CN)_3_ is the final product, reaction 13. DFT calculations at the M06/6-31++G(d,p) level of theory show that the unpaired electron localizes in the π* orbital of acetonitrile, resulting in the bent CH_3_CN^–^ radical. Proton transfer leads to the [CH_3_CN,H] product, which leaves the cluster.

Efficient reactions are also in this case observed with reactants that undergo hydrogen bonding, like CH_3_CN, or ionic dissolution in the water cluster, like HCl. For these reactants, the position of the electron in the cluster seems irrelevant. O_2_ and CO_2_, on the other hand, react with relatively small rate coefficients, which is attributed to a significant steric factor, i.e., the reaction only proceeds if the neutral molecule hits the cluster surface in the vicinity of solvated electron.

#### 2.3.4 Reactivity of Hydrated Monovalent Transition Metal Ions

Given that + I oxidation state does not commonly occur in aqueous solution for M = Cr, Mn, Fe, Co, Ni, Zn, one may expect that these singly charged metal centers in water clusters M^+^(H_2_O)_*n*_ are easily oxidized and behave largely like Mg^+^(H_2_O)_*n*_. Experiments with HCl, however, revealed that these metals react quite differently. Earlier experiments of Ag^+^(H_2_O)_*n*_ reacting with HCl^160^ indicated that precipitation reactions occur on the single molecule level in gas-phase clusters. *Ab initio* molecular dynamics simulations of a Ag^+^ and Cl^–^ ion in a water cluster corroborated this interpretation, resulting in a AgCl molecule that moves to the cluster surface.^161^ Comparison with the results for M = Ag^+^ indicates that most transition metals undergo a precipitation reaction, forming an intact MCl molecule in the water cluster.^162,163^Reaction 14 was observed for M = Cr, Mn, Fe, Co, Ni, Cu, Ag.^162^
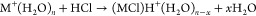
14For zinc, the situation is more complex. Only uptake of a second HCl molecule results in the elimination of an H atom and oxidation of the metal center.^83^ Despite the high solubility of ZnCl_2_, the final stage of the reaction suggests that either an intact ZnCl_2_ molecule or a ZnCl^+^ molecular ion has precipitated in the cluster. The smallest ions observed after 35 s are ZnCl^+^(H_2_O)_*n*_, *n* = 3,4.

The reactions of M^+^(H_2_O)_*n*_ with nitric oxide were studied for M = V, Cr, Mn, Fe, Co, Ni, Cu, Zn with *n* ≤ 40.^84^ Chromium, cobalt, and nickel containing clusters undergo ligand exchange, without any hint of further rearrangements. While chromium reacts with up to four NO molecules, cobalt takes up two and nickel only one. The uptake of the third and fourth NO molecule by Cr^+^(H_2_O)_*n*_, however, happens only for small clusters, which feature empty coordination sites at the metal center. For cobalt and nickel, the uptake accelerates over time with the shrinking of the hydration shell due to BIRD, which suggests that NO requires access to a surface or near-surface solvated metal center in order to stay in the cluster.

Redox chemistry in larger clusters is observed for iron and zinc. Here, one NO molecule is taken up, followed by elimination of HNO and formation of a hydrated metal hydroxide, reaction 15. HNO elimination is most efficient in the size regime around *n* = 15–20, suggesting that the reaction requires a certain degree of hydration and at the same time a metal center at or near the cluster surface.
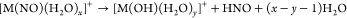
15Our experiments suggest that the formation of the hydroxide is a unimolecular process. It is noticeable that the region of highest reactivity, *n* ≈ 15–20, is comparable to the size region where Al^+^(H_2_O)_*n*_ clusters form H_2_ most efficiently.^164^ This indicates that a delicate balance between hydration and structural flexibility is required to afford the concerted proton transfers that are most likely involved in both reactions. For manganese, HNO formation is observed only for HMnOH^+^(H_2_O)_*n*_, *n* ≤ 4, where the reactant has direct access to the hydride ligand in the hexacoordinated complex. In such small clusters, however, it does not really make sense to discuss surface vs internal solvation.

Uptake of CO_2_ and O_2_ is less efficient than that of NO.^11^ Very slow uptake of CO_2_ is observed for Co^+^(H_2_O)_*n*_, and slow uptake for Cr^+^(H_2_O)_*n*_. Both hydrated ions show a relatively fast uptake of O_2_, and also Zn^+^(H_2_O)_*n*_ and Ni^+^(H_2_O)_*n*_ react with O_2_, the latter albeit more slowly. For chromium and nickel, the reaction accelerates with shrinking size, while for cobalt, the rate somewhat decelerates. Steric access to the metal ion and subtle changes in the thermochemistry with changing cluster size are probably responsible for this size-dependent reactivity. For iron, only clusters in the range of 3–4 water molecules undergo ligand exchange with O_2_.

Reductive decomposition of the greenhouse gas nitrous oxide^165,166^ by gas-phase monovalent metal ions M^+^ has been investigated by inductively coupled plasma/selected-ion flow tube mass spectrometry.^167,168^ Microsolvation in hydrated clusters M^+^(H_2_O)_*n*_ is expected to change this reactivity considerably.^5^ In our FT-ICR studies, among the monovalent first-row transition metal ions, only Co^+^(H_2_O)_*n*_, *n* ≈ 4–35, showed reactivity.^11^ Co^+^(H_2_O)_*n*_ clusters reduce N_2_O and form either a cobalt(II), [CoOH]^+^(H_2_O)_*n*_, or a cobalt(III), [CoO]^+^(H_2_O)_*n*_, species. These two competing reactions are strongly size-dependent, with formation of the Co(III) observed only in a size region of ⟨*n*⟩ ≤ 20.

**Scheme 1:**
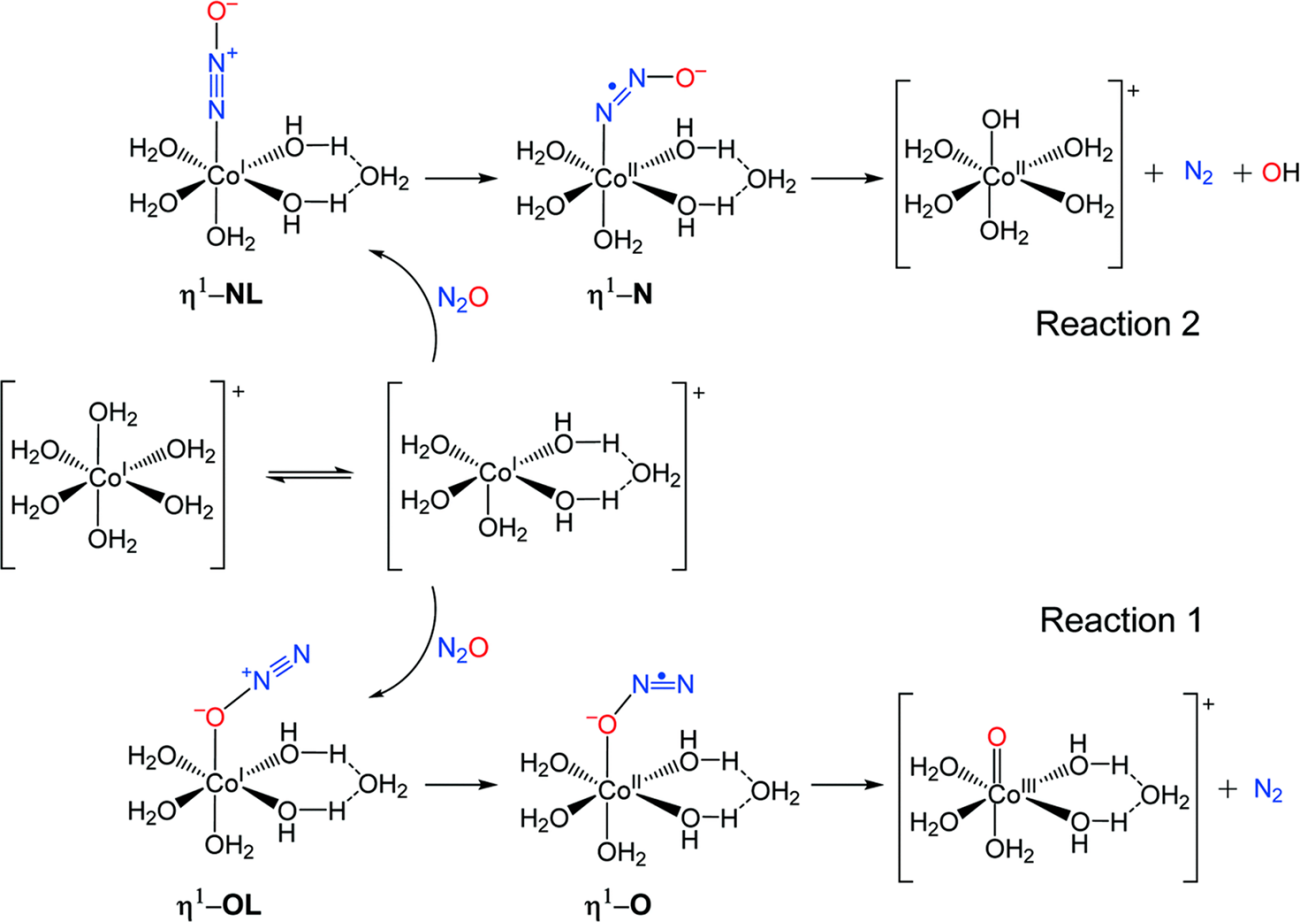
Decomposition of N_2_O on Co(H_2_O)_*n*_^+^ through Formation of Either Co–O or Co–N Bond (Reactions 1 and 2, Respectively) Reproduced with permission from ref (169). Copyright 2021, Royal Society of Chemistry.

**Figure 10:**
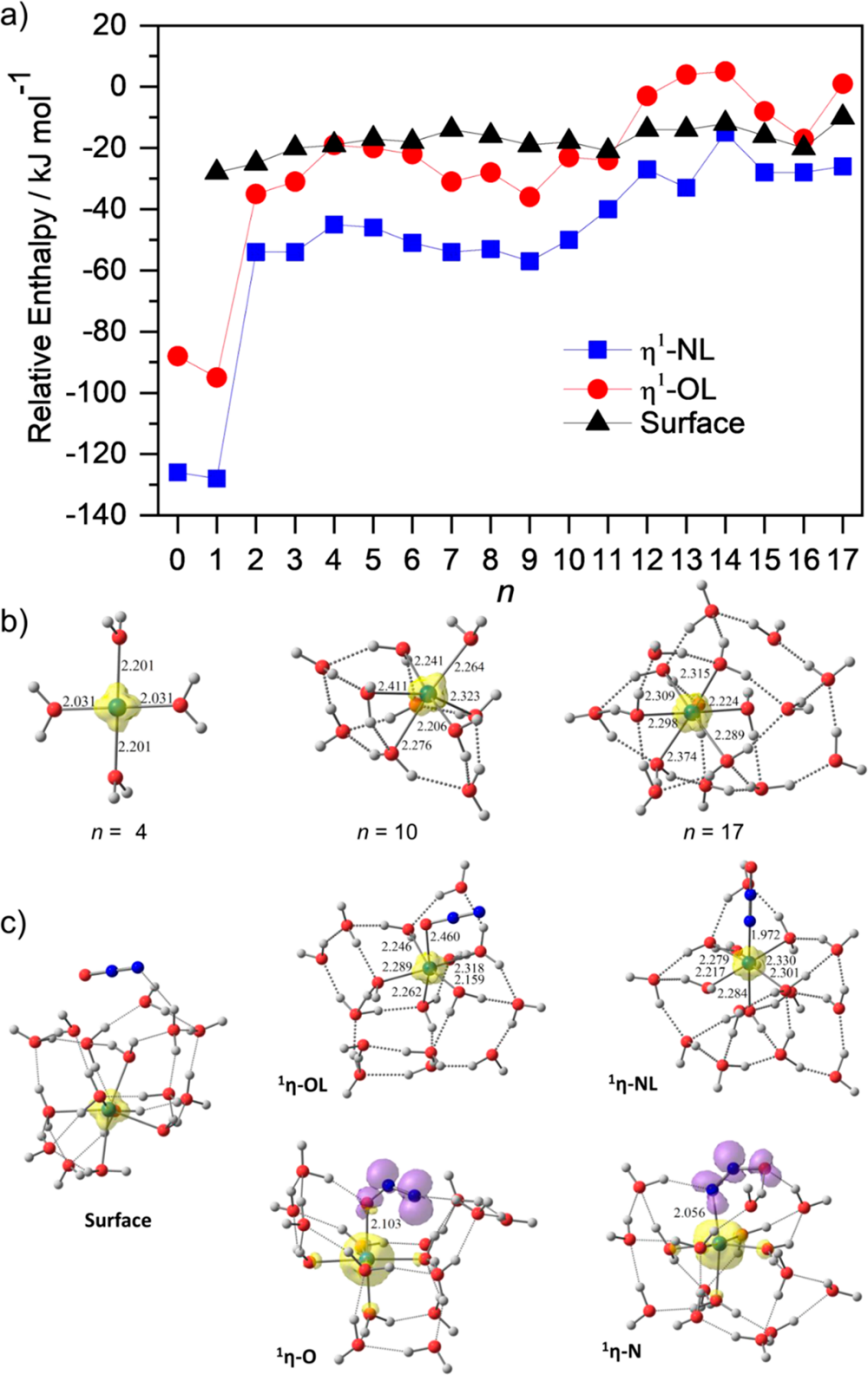
(a) Binding energies of N_2_O toward Co^+^(H_2_O)_*n*_ with different binding modes (^**1**^**η-NL**, ^**1**^**η-OL**, and surface binding) calculated at the M06/6-311++G(d,p) level of theory. (b) Lowest-lying geometries of Co^+^(H_2_O)_*n*_, for *n* = 4, 10, and 17. (c) Lowest-lying geometries of [(Co^+^)(N_2_O)](H_2_O)_*n*_ (^**1**^**η-NL** and ^**1**^**η-OL**) and [(Co^2+^)(N_2_O^–^)](H_2_O)_*n*_ (^**1**^**η-N** and ^**1**^**η-O**), for *n* = 16. The yellow and purple clouds illustrate the alpha and beta spin densities, respectively, with an isovalue of 0.06 au. Reproduced and adapted with permission from ref (169). Copyright 2021, Royal Society of Chemistry.

The formation of [CoO]^+^(H_2_O)_*n*_ presumably involves an attack of N_2_O toward Co^+^ to initially form an O-bound [Co-ONN]^+^ core (^**1**^**η-OL**), which will then undergo electron transfer to yield [(Co^2+^)(^−^ONN)] (^**1**^**η-O**) followed by an O–N bond cleavage to liberate N_2_ (reaction 1, Scheme 1). This straightforward mechanism is commonplace for typical metal-mediated N_2_O decompositions.^170,171^ By contrast, the formation of [CoOH]^+^(H_2_O)_*n*_ is surprising and likely governed by the detailed solvation structures of the ionic core in the hydrated clusters with varying sizes. A recent theoretical study employing DFT at the M06/6-311++G(d,p) level of theory suggests that [CoOH]^+^(H_2_O)_*n*_ is formed through an N-bound [Co-NNO]^+^ (^**1**^**η-NL**) core followed by simultaneous losses of N_2_ and OH through an electron-transferred intermediate [(Co^2+^)(NNO^–^)] (^**1**^**η-N**) (reaction 2, Scheme 1).^169^ The selectivity of these reactions depends on the subtle trends of binding modes of N_2_O toward Co^+^(H_2_O)_*n*_ with increasing cluster size *n*. Figure 10a shows calculated binding energies for N_2_O coordinating to cobalt as well as for a surface-bound N_2_O molecule, which does not form a bond to the metal center. In Figure 10b, hydrated cobalt ion geometries without N_2_O reactant are shown, while Figure 10c provides the most important structures of Co^+^(H_2_O)_16_(N_2_O).

Theory predicts that for Co^+^(H_2_O)_*n*_ with *n* ≤ 4, water molecules successively add to the first solvation shell of the Co^+^ center to form di-, tri- and tetra-coordination (2c, 3c, and 4c, respectively). The 4c (square-planar) geometry of Co^+^ remains as the lowest-energy coordination up to *n* = 9, beyond which the internally solvated penta- and hexa-coordinations (5c and 6c, respectively) become energetically more favorable (Figure 10b). Reaction of N_2_O toward the clusters will initially form the weakly bound surface-solvated geometries with binding energies of about −18 ± 4 kJ mol^–1^ (Figure 10a).

Unlike the hydrated Mg^+^ ion, of which the actual reductive properties depend primarily on its 3s electron solvated out into the water clusters,^150,151,155,159^ the reduction of N_2_O in the surface-solvated state of the hydrated Co^+^ is inefficient. Instead, the redox reaction will happen only after N_2_O anchors directly to the Co^+^ center through the O-bound (^**1**^**η-OL**) or the N-bound (^**1**^**η-NL**) binding motif (Figure 10c). While ^**1**^**η-OL** will follow Reaction 1 in Scheme 1 to form the expected [CoO]^+^(H_2_O)_*n*_, its binding energy is comparable to, or even smaller than, that of the surface-solvated state, especially for large cluster sizes. These theoretical results imply that even if the energetically unfavorable ^**1**^**η-OL** isomers are initially formed, the weakly O-bound N_2_O will easily detach from Co^+^ to the cluster surface and then reapproach to the metal center through its N atom to form the stronger-bound ^**1**^**η-NL**, followed by the formation of the unexpected [CoOH]^+^(H_2_O)_*n*_ via Reaction 2 in Scheme 1. Detailed mechanistic examination suggests that Reaction 1 is kinetically controlled by the initial electron-transfer process to form ^**1**^**η-O**, which is likely attributed to the insufficient solvation of the anionic oxygen atom of the reduced N_2_O^–^ that is anchored internally to the metal center. On the other hand, the anionic oxygen atom of ^**1**^**η-N** is pointing away from the ionic core and undergoes flexible hydrogen bonding with water molecules, and the N–O bond cleavage is always rate-determining for Reaction 2 (Scheme 1). Since the electron-transfer step involving charge separation is more sensitive to hydration than the N–O bond cleavage step, it is reasonable that Reaction 1 becomes less competitive than Reaction 2 with increasing cluster sizes (Scheme 1). However, when the ionic core is further submerged in very large clusters, the reactivity vanishes completely. This example illustrates the complexity of surface effects, even at relatively large cluster sizes.

With acetonitrile, M^+^(H_2_O)_*n*_, M = Cr, Mn, Fe, Co, Ni, Zn, reacts by ligand exchange, taking up several CH_3_CN molecules without apparent size dependence for the first reaction steps.^85^ Interestingly, Zn^+^(H_2_O)_*n*_ clusters exhibit a behavior that is intermediate between the other first row transition metals and Mg^+^(H_2_O)_*n*_: Ligand exchange competes with the oxidation reaction 12, and formation of the hydroxide may still occur if acetonitrile molecules are already present. It is not clear whether this is a delayed intracluster reaction or triggered by a collision with another CH_3_CN molecule. Quantum chemistry shows that both electron transfer from Zn^+^ to CH_3_CN as well as subsequent proton transfer to eliminate [CH_3_CN,H] face barriers, and the reaction is overall near thermoneutral for larger clusters. While the CH_3_CN uptake proceeds without apparent size dependence, formation of hydroxide species seems favored for larger clusters, evidenced by the average cluster size of reactant and products.

Upon increasing the size of the reactant, the complexity also increases. Reactions of M^+^(H_2_O)_*n*_ with 1-iodopropane, C_3_H_7_I, provide a range of reaction products, which are specific for certain metals.^86^ The most redox-active metals are again Cr, Co and Zn, which react to form the metal iodide, with little size dependence, reaction 16.
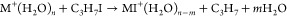
16

Ligand exchange is observed for all cluster sizes of Cu^+^(H_2_O)_*n*_, but only with small clusters for M = Cr, Mn, Fe, Co, Ni. For iron and manganese, traces of the metal iodide are also observed for small clusters. In the final stages of the reaction of the cobalt and nickel species, HI elimination is observed from clusters containing several iodopropane molecules. Zn^+^(H_2_O)_*n*_, on the other hand, reacts efficiently with formation of ZnI^+^(H_2_O)_*m*_, with a slight acceleration for smaller clusters. This behavior ties in nicely with the IRMPD results discussed above, which indicate that Zn^+^ remains on the cluster surface even for larger clusters. For hydrophobic reactants like iodopropane, surface or near-surface solvation definitely increases reactivity.

As discussed above, reactions with HCl or CH_3_CN, which interact strongly with the hydrogen bonded network, proceed efficiently, and it is not relevant whether the charge center is surface or internal solvated. The situation is different for reactants that interact weaker with the water cluster. Accelerated rates for smaller clusters have been observed for hydrated Co^+^ and Ni^+^ with NO, Cr^+^ and Ni^+^ with O_2_, and Co^+^ with N_2_O. The rate coefficients reflect a delicate balance of access to the metal center and size-dependent shifts in the thermochemistry, which plays out quite individually for each combination of metal and neutral reactant. Very complex, size dependent reactions occur with C_3_H_7_I, the largest reactant studied so far. Here the reaction with Zn^+^(H_2_O)_*n*_ proceeds efficiently for all cluster sizes, in line with the surface solvation of Zn^+^ inferred from IRMPD experiments.

## 3 Conclusions

Surface or asymmetric solvation of soft ions can be rationalized by looking at the emerging solvation shell. Two factors determine cluster growth, maximizing hydrogen bonding and maximizing electrostatic interaction with the ion. The first water molecule inevitably binds to the ion, but each additional water molecule has the choice between either binding to the ion or to integrate into the hydrogen bonded network of the previous water molecules, without direct contact to the ion. As long as the latter is energetically favorable, the charge center remains at the cluster surface.

Electronic spectra of hydrated metal ions, however, are very sensitive to the structure of the first solvation shell. Strong redshifts have been observed with increasing coordination number for V^+^(H_2_O)_*n*_ and Al^+^(H_2_O)_*n*_. Hydrated electrons are more sensitive to the size of the water cluster up to 100 water molecules, and two binding motifs were identified in the electronic spectra that represent varying degrees in the transition from surface to internal solvation. Overall, electronic spectroscopy is very sensitive to surface vs internal solvation. Moreover, the studied examples illustrate that the transition from surface to internal solvation may proceed rather gradually and that these terms are actually a very poor description of the subtle structural changes that occur in hydrated ions.

Infrared spectroscopy in combination with quantum chemical calculations is a valuable tool for the structural characterization of hydrated ions, and the surface solvation of Zn^+^ and Zn_2_^+^ in small water clusters could be identified. With respect to surface vs internal solvation, however, it is not always specific since the band position of the surface solvated molecular ion CO_2_^–^(H_2_O)_20_ is almost identical with the value from bulk aqueous solution. Also H/D exchange reactions are not really helpful in determining surface vs internal solvation.

Nanocalorimetry shows that the thermochemistry of chemical reactions in water clusters is quite compatible with bulk aqueous solution, with error limits in the energy range of one hydrogen bond, or 20 kJ mol^–1^. This may be in part due to a cancellation of the differences between cluster and bulk hydration enthalpies of reactant and product clusters, but all the evidence gathered suggests that this difference is small for clusters beyond a size of 50 water molecules. The influence of surface vs internal solvation is smaller than the error limits of the method, and no clear effect was identified so far.

Ion–molecule reactions of hydrated ions with neutral reactants like HCl or CH_3_CN, which interact strongly with the hydrogen bonded network or even undergo ionic dissolution, proceed irrespective of surface vs internal solvation of the charge center. However, if the neutral reactant interacts only weakly with the hydrogen bonded network, the initial uptake will be influenced by surface vs internal solvation. While in the bulk, diffusion ensures intimate contact between dissolved gases and hydrated ions, a weakly interacting molecule in the gas phase may simply bounce off the cluster surface, unless it accidentally impacts at or near the reactive center. This leads to a pronounced steric factor, along with an acceleration in reactivity with decreasing cluster size. A very nice example is CO_2_^–^(H_2_O)_*n*_ reacting with O_2_, which proceeds via a CO_4_^–^ intermediate. Collisions of Co^+^(H_2_O)_*n*_ with N_2_O are reactive only for *n* ≈ 4–35. Zn^+^(H_2_O)_*n*_ reacts efficiently with C_3_H_7_I, since Zn^+^ is exposed at the surface. Particularly subtle size dependences are observed if concerted proton transfer is involved, e.g., in the HNO elimination in the reactions of NO with Fe^+^(H_2_O)_*n*_ and Zn^+^(H_2_O)_*n*_. For these more complex rearrangements, however, the question of surface vs. internal hydration is too simplified.

In the fewest possible words, electronic spectra are very sensitive to the position of the ion, and the same holds true for bimolecular reactions with neutrals that do not integrate into the hydrogen bonded network of the water cluster. On the other hand, for reactions with strongly interacting neutral molecules, it does not really matter where the ion is located, since the neutral reactant is roaming around the water cluster. The effect on the thermochemistry of ion–molecule reactions is also relatively small. However, whether an ion in a gas-phase water cluster is surface or internally solvated remains an intriguing problem worthwhile of investigation.

## Dedication

Dedicated to Professor Peter B. Armentrout on the occasion of his 70th birthday.
